# Overexpression of *NtCBL5A* Leads to Necrotic Lesions by Enhancing Na^+^ Sensitivity of Tobacco Leaves Under Salt Stress

**DOI:** 10.3389/fpls.2021.740976

**Published:** 2021-09-17

**Authors:** Jingjing Mao, Jiaping Yuan, Zhijie Mo, Lulu An, Sujuan Shi, Richard G. F. Visser, Yuling Bai, Yuhe Sun, Guanshan Liu, Haobao Liu, Qian Wang, C. Gerard van der Linden

**Affiliations:** ^1^Tobacco Research Institute, Chinese Academy of Agricultural Sciences (CAAS), Qingdao, China; ^2^Graduate School of Chinese Academy of Agricultural Sciences (GSCAAS), Beijing, China; ^3^Department of Plant Breeding, Wageningen University & Research (WUR), Wageningen, Netherlands; ^4^Graduate School of Experimental Plant Sciences, Wageningen University, Wageningen, Netherlands; ^5^School of Life Science and Engineering, Lanzhou University of Technology, Lanzhou, China

**Keywords:** CBL PROTEIN, Na^+^, immune response, necrotic lesions, photosystem, reactive oxygen species, salt stress, tobacco

## Abstract

Many tobacco (*Nicotiana tabacum*) cultivars are salt-tolerant and thus are potential model plants to study the mechanisms of salt stress tolerance. The CALCINEURIN B-LIKE PROTEIN (CBL) is a vital family of plant calcium sensor proteins that can transmit Ca^2+^ signals triggered by environmental stimuli including salt stress. Therefore, assessing the potential of *NtCBL* for genetic improvement of salt stress is valuable. In our studies on *NtCBL* members, constitutive overexpression of *NtCBL5A* was found to cause salt supersensitivity with necrotic lesions on leaves. *NtCBL5A*-overexpressing (OE) leaves tended to curl and accumulated high levels of reactive oxygen species (ROS) under salt stress. The supersensitivity of *NtCBL5A*-OE leaves was specifically induced by Na^+^, but not by Cl^−^, osmotic stress, or drought stress. Ion content measurements indicated that *NtCBL5A*-OE leaves showed sensitivity to the Na^+^ accumulation levels that wild-type leaves could tolerate. Furthermore, transcriptome profiling showed that many immune response-related genes are significantly upregulated and photosynthetic machinery-related genes are significantly downregulated in salt-stressed *NtCBL5A*-OE leaves. In addition, the expression of several cation homeostasis-related genes was also affected in salt-stressed *NtCBL5A*-OE leaves. In conclusion, the constitutive overexpression of *NtCBL5A* interferes with the normal salt stress response of tobacco plants and leads to Na^+^-dependent leaf necrosis by enhancing the sensitivity of transgenic leaves to Na^+^. This Na^+^ sensitivity of *NtCBL5A*-OE leaves might result from the abnormal Na^+^ compartmentalization, plant photosynthesis, and plant immune response triggered by the constitutive overexpression of *NtCBL5A*. Identifying genes and pathways involved in this unusual salt stress response can provide new insights into the salt stress response of tobacco plants.

## Introduction

Soil salinity causes serious yield losses because of its widespread occurrence and severe effects on crop physiology and metabolism (Munns and Tester, 2008). The potential crop yield losses induced by moderate salinity (8~10 dS/m) are about 55, 28, and 15% in corn, wheat, and cotton, respectively (Satir and Berberoglu, 2016). It is estimated that about 30% of irrigated lands are salt-affected and thus commercially unproductive (Zaman and Heng, 2018). There are several strategies for the utilization of salt-affected lands, one of which is to breed or genetically engineer new varieties suitable for saline soils (Gong et al., 2020). Therefore, understanding the mechanisms underlying the plant salt stress response is of fundamental importance to mitigating the negative impact of soil degradation.

Salt stress can be divided into two phases, the first of which is the osmotic or water-deficit stress (Munns, 2005; Van Zelm et al., 2020). When exposed to salinity stress, plants experience an immediate osmotic effect around root cells that impairs water uptake and disturbs associated cell growth and metabolism, similar to the effects of drought stress. The second phase is a salt-specific ion toxicity effect (Munns, 2005; Van Zelm et al., 2020). At prolonged exposure to salinity, Na^+^ and Cl^−^ are transported to leaf blades by the transpiration stream. When the ions accumulate to high levels, they become toxic and cause damage (Munns and Tester, 2008). The osmotic stress can be measured as a rapid inhibition of the rate of expansion of young leaves, while ion toxicity causes stress-induced senescence of older leaves due to either high leaf Na^+^ concentrations or low tolerance of the accumulated Na^+^ (Munns and Tester, 2008).

Plants respond to the two phases of salinity stress *via* different signaling pathways (Bartels and Sunkar, 2005). Ca^2+^ functions as a secondary messenger to couple a wide range of extracellular stimuli to intracellular responses (Snedden and Fromm, 1998, 2001). Osmotic and salt stresses induce rapid [Ca^2+^]_cyt_ transients in the cytosol that trigger downstream pathways, allowing plants to adapt to these environmental changes by regulating enzymatic activity, ion channel activity, and gene expression (Knight and Knight, 2001; Snedden and Fromm, 2001). The transduction of Ca^2+^ signals depends on Ca^2+^-sensor proteins (Trewavas and Malhό, 1998). To date, four major classes of Ca^2+^ sensor proteins have been characterized in plants: CALMODULIN (CaM), CALMODULIN-LIKE PROTEIN (CML), CALCIUM-DEPENDENT PROTEIN KINASE (CDPK), and CALCINEURIN B-LIKE PROTEIN (CBL; Perochon et al., 2011).

As vital Ca^2+^-sensors, CBLs mainly function by regulating the kinase activity of their partners CBL-INTERACTING PROTEIN KINASEs (CIPKs) in response to various abiotic stresses including salt stress (Luan, 2009). So far, several CBLs from various plant species have been reported to participate in the salt stress response by facilitating Na^+^ exudation and vacuolar sequestration. In the root, *Arabidopsis thaliana* AtCBL4 interacts with AtCIPK24 to unlock its kinase activity, and the activated AtCIPK24 can phosphorylate and activate the plasma membrane-localized Na^+^/H^+^ antiporter SALT OVERLY SENSITIVE 1 (SOS1), leading to Na^+^ extrusion from root cells (Qiu et al., 2002; Shi et al., 2002; Gong et al., 2020). This so-called SOS pathway was found to be conserved in many other plant species, including rice (*Oryza sativa*), poplar (*Populus trichocarpa* and *Populus euphratica*), mustard (*Brassica juncea*), and apple (*Malus domestica*; Martinez-Atienza et al., 2007; Tang et al., 2010; Chakraborty et al., 2012; Hu et al., 2012; Lv et al., 2014). In the shoot, AtCBL10 is capable of interacting with both AtCIPK24 and AtCIPK8 to phosphorylate SOS1, and thus enhancing Na^+^ efflux from cells (Quan et al., 2007; Lin et al., 2009; Yin et al., 2019). Moreover, the AtCBL10-AtCIPK24 complex was suggested to activate the tonoplast-localized Na^+^/H^+^ EXCHANGER (AtNHX) to sequester Na^+^ into vacuole for salt storage and detoxification of the cytosol (Qiu et al., 2004; Kim et al., 2007). Orthologs of AtCBL10 in other species including wild tobacco (*Nicotiana sylvestris*), tomato (*Solanum lycopersicum*), and poplar (*P. trichocarpa* and *P. euphratica*) were also reported to be involved in the Na^+^ tissue tolerance mechanism (Li et al., 2012a; Tang et al., 2014; Dong et al., 2015; Egea et al., 2018). There are other CBLs that may participate in the salt stress response, although their roles and mechanisms remain to be elucidated. For instance, both *AtCBL5*- and *AtCBL1*-overexpressing (OE) plants showed enhanced tolerance to salt stress (Cheong, 2003; Cheong et al., 2010). Ectopic and constitutive expression of *CBL1* orthologs from the epiphytic orchid (*Sedirea japonica*), rape (*Brassica napus*), and soybean (*Glycine max*) in *Arabidopsis* also enhanced salt tolerance (Chen et al., 2012; Li et al., 2012b; Cho et al., 2018). However, the ectopic expression of *PeCBL1* in *Arabidopsis* led to salt sensitivity, with the transgenic lines not being able to exclude Na^+^ under saline conditions (Zhang et al., 2013).

Tobacco (*Nicotiana tabacum*) has been investigated as a potential model crop to adapt to salt stress *via* various strategies (Sun et al., 2020). *Nicotiana tabacum* L. cv. Zhongyan 100 is of good salt tolerance, which can survive under 300mM NaCl in a hydroponic growth system (data not shown). In our studies on *NtCBL* members in Zhongyan 100, *NtCBL5A* attracted our attention because its overexpression broke the salt tolerance of Zhongyan 100 and led to salt supersensitivity with severe necrotic lesions on leaves. Studies on *CBL5* orthologs were only reported in *Arabidopsis*: overexpression of *AtCBL5* enhanced the salt tolerance of transgenic *Arabidopsis* (Cheong et al., 2010; Wang et al., 2013). In this study, we explored the mechanisms underlying the salt sensitivity of *NtCBL5A*-OE leaves at the physiological, biochemical, and molecular levels. Our results indicate that constitutive overexpression of *NtCBL5A* leads to Na^+^-dependent leaf necrosis by enhancing the sensitivity of transgenic tobacco leaves to Na^+^. This Na^+^ sensitivity may be related to Na^+^ compartmentalization, plant photosynthesis, and plant immune response.

## Materials and Methods

### Plant Material

*Nicotiana tabacum* L. cv. Zhongyan 100 was obtained from the Tobacco Research Institute of CAAS.

### Plasmids Construction

The coding sequence of *NtCBL5A* was first amplified from cDNA synthesized from RNA extracted from veins of tobacco cultivar Zhongyan 100 with primers NtCBL5A-1F and NtCBL5A-1R (Supplementary Table S1) and cloned into the pMD19-T vector for sequencing. The *NtCBL5A* CDS was amplified from pMD19-T-NtCBL5A with primers NtCBL5A-3F-*Sac*Iand NtCBL5A-3R-*Kpn*I (Supplementary Table S1). The gel-purified amplicon was digested with *Sac*Iand *Kpn*Iand cloned into the pCHF3 vector, resulting in the binary recombinant vector pCHF3-NtCBL5A. To construct the promoter plasmid pBI101-ProNtCBL5A::GUS, the upstream sequence of the *NtCBL5A* gene was identified from scaffold Nsyl_scaffold38441 of the tobacco genome. The 2,780bp upstream regulatory region of *NtCBL5A* including promoter sequence (ProNtCBL5A; Supplementary Figure S1B) was amplified from Zhongyan100 DNA using the primer pair NtCBL5Apro-1F-*Sal* Iand NtCBL5Apro-1R-*Sma*I (Supplementary Table S1) and cloned into a pMD19-T vector for sequencing. The promoter segment was subcloned from the pMD19-T-ProNtCBL5A construct using *Sal*Iand *Sma*Iinto pBI101. All the constructs were confirmed by DNA sequencing by the BGI company. Confirmed constructs were transformed into *Agrobacterium tumefaciens* EHA105 for tobacco transformation.

### Generation of Transgenic Plants

To generate the *NtCBL5A*-OE lines and *ProNtCBL5A::GUS* transgenic plants, *Agrobacterium* carrying pCHF3-NtCBL5A plasmid and pBI101-ProNtCBL5A::GUS plasmid were introduced into Zhongyan 100, respectively, by the *Agrobacterium*-mediated leaf disk transformation method (Horsch et al., 1985). To screen for *NtCBL5A*-OE lines, positive transgenic plants of the T0 generation were identified by PCR with the primers NtCBL5A-1F and pCHF3-R (Supplementary Table S1). The overexpression levels of all positive plants were checked by Real-time quantitative PCR (RT-qPCR), the RNA samples for RT-qPCR were isolated from mixed whole plants at 12days after germination (DAG) under control conditions. Eight lines with high *NtCBL5A* overexpression of the original 17 transgenic lines (T0 generation) were selected for propagation. More than 200 T1 seeds from T0 plants with high gene overexpression levels were harvested and subsequently screened on the selection medium (1/2 MS medium with 50μg/ml kanamycin). All these lines show sensitivity to salt stress. Two T1 lines (OE-2 and OE-15) with a segregation of around 3:1 (tolerance: sensitivity) were selected for harvesting T2 seeds. More than 200 T2 seeds were screened on the selection medium. The T2 generation with 100% kanamycin resistance was considered as homozygous plants and used for the evaluation of stress tolerance. Positive *ProNtCBL5A::GUS* transgenic lines of the T0 generation were identified by PCR with the primers pBI101-F and NtCBL5Apro-1R-*Sma*I (Supplementary Table S1). More than 200 seeds from T0 seedlings were harvested and subsequently screened on selection medium as described above and positive T1 plants from three independent lines were selected for GUS staining assay.

### Application and Phenotyping of Salt and Drought Treatment

Salt and drought stress experiments were conducted in 2019 at Unifarm, Wageningen University & Research in the Netherlands. Conditions of the greenhouse were 16h light/8h dark at 25/23°C and 70% relative humidity. The shortwave radiation level was maintained in the greenhouse compartment using artificial photosynthetically active radiation (PAR) when the incoming shortwave radiation was below 200 Wm^−2^.

For the salt tolerance evaluations, tobacco seeds were sown in soil, and they germinated after about 8days. About at 12DAG, the young seedlings were transplanted in rock-wool plugs within float trays for 8days, then they were transplanted to a circular flow hydroponic system filled with 1/2 Hoagland’s nutrient solution (500L). The water used to prepare 1/2 Hoagland’s nutrient solution contained trace amounts of Na^+^ and Cl^−^ (5.04 and 6.72μg/ml, respectively). After 6days of acclimatization (at ~30 DAG), NaCl was added to the nutrient solution to a concentration of 50mM on the first day to avoid salt shock, and the final NaCl concentration of 100mM NaCl was reached the next day. From 4days after the start of the treatment (DAT), photographs of the plants were taken every day until harvest. For assessing salt tolerance, indicative traits such as growth traits (leaf width and length, root length, and fresh/dry biomass) and ion content were measured.

For the drought stress evaluation, a pilot experiment was conducted first to determine the wilting point of tobacco under the greenhouse conditions. Tobacco seeds were sown in soil. Young seedlings were transplanted to trays and pots at 12 and 22 DAG. Sixteen days after transplantation to pots (38DAG), watering was stopped until the leaves started to exhibit slight wilting, at a recorded Soil Water Content (SWC) of 28% Full Field Capacity (FFC; ~43 DAG). For the drought tolerance evaluation experiment, SWC was kept around 60% FFC (control conditions) and 28% FFC (drought conditions) by supplying a limited amount of water every day for 3weeks (~64DAG). The SWC was monitored and recorded with a Grodan Water Content Meter and a gravimetrical method, where SWC=(W_wet_−W_dry_)/FFC*100% (W_wet_ is the weight of wet soil and W_dry_ is the weight of dry soil). For assessing salt tolerance, shoot biomass and chlorophyll content were measured. Chlorophyll content was measured with the Minolta SPAD 502 Chlorophyll Meter (Ling et al., 2011). For chlorophyll content values, the average was taken of the measurements of five different areas of the eigth leaf (the first new leaf that appeared after the drought treatment started).

### Ion and Osmotic Stress Evaluation

For the ion and osmotic stress evaluation experiments, the plants were prepared as described for the salt evaluation experiments. The experiments were conducted in a hydroponic system filled with 1/2 Hoagland’s nutrient solution in 3L containers. The solution was refreshed every 2days to keep enough nutrition and a stable stress treatment. After 6days of acclimatization, the stress was built up in two steps: ion concentrations were raised to 50mM NaCl/NaNO_3_/KNO_3_/KCl, and for the osmotic stress treatment to 8% PEG6000, and the final stress conditions were reached 100mM NaCl/NaNO_3_/KNO_3_/KCl, 15% PEG6000 at the next day. Photographs of the plants were taken at 9DAT.

### Ion Content Measurement

Ion content measurement contained three biological replications and every biological replication is a pool of three plants. Root samples were rinsed in ddH_2_O first, and then were dried with absorbent paper to get rid of ions on the outside of the roots. Fresh samples were dried at 105°C until stable weights, and then the dry tissue was crushed to powder with a grinder. About 30~50mg of dry sample was placed into a test tube. The powdered samples were ashed at 650°C for 6h. One milliliter of 3M formic acid was added into the test tube and was shaken for 20min at 5,000rpm at 99.9°C. Then 9ml Milli-Q® was added into the test tube and mixed. Then 0.2ml sample was taken out and added into 9.8ml Milli-Q® for 50 times dilution. The ion contents of dilution samples were measured using the Ion Chromatography (IC) system 850 Professional (Metrohm Switzerland).

### GUS and DAB Staining

GUS staining was conducted using β-Galactosidase Reporter Gene Staining Kit (Beijing Leagene Biotech. Co., Ltd., Cat No./ID: DP0013, Beijing, China). Samples were placed into GUS staining solution and incubated at 37°C overnight. The tissues then were placed in 95% ethanol until chlorophyll was washed out, and samples were photographed. The tissues were stored in Formaldehyde-Acetic Acid-Ethanol (FAA) Fix Solution (Wuhan Servicebio Technology Co., Ltd., Cat No./ID: G1103-500 ML, Wuhan, China). For H_2_O_2_ visualization with DAB staining, leaves were put in 1mg/ml DAB (3, 3'-Diaminobenzidine tetrahydrochloride hydrate; Sigma-Aldrich, Cat No./ID: D5637-1G, Darmstadt, Germany) and vacuum infiltrated until DAB solution was taken up, after which the leaves were incubated in DAB staining solution for 16h in the dark. The leaves were subsequently transferred to 95% ethanol for 24h to remove chlorophyll. Afterward, leaves were photographed (Kissoudis, 2016).

### RNA Isolation and RNA-seq Analyses

At 4DAT, the fifth leaf blades (with main veins removed) of four individual plants under control conditions and saline conditions in the daytime were sampled and frozen immediately in liquid nitrogen. Leaf blades from four individual plants were mixed and ground to powder. RNA was isolated and purified with the RNeasy Plus Mini Kit (Qiagen, Cat No./ID: 74134, the Netherlands) following the manufacturer’s protocol. RNA samples of WT and OE-2 overexpression lines from two independent salt treatment experiments were used for transcriptome sequencing. RNA integrity was assessed using the RNA Nano 6000 Assay Kit of the Bioanalyzer 2100 system (Agilent Technologies, CA, United States). RNA-seq was performed by the Novogene using Illumina Polymerase-based sequencing-by-synthesis, obtaining a read length of 150bp and coverage of 48–81 million reads per sample. Raw reads of fastq format were firstly processed through in-house perl scripts so that all the downstream analyses were based on the clean data with high quality. Reference genome and gene model annotation files^1^ were downloaded from NCBI. Index of the reference genome was built using Hisat2 v2.0.5 and paired-end clean reads were aligned to the reference genome using Hisat2 v2.05. The mapped reads of each sample were assembled by StringTie (v1.3.3b; Pertea et al., 2015) in a reference-based approach. FeatureCounts v1.5.0-p3 was used to count the reads numbers mapped to each gene and then FPKM of each gene was calculated based on the length of the gene and read counts mapped to this gene. Differentially expressed genes (DEGs) of two groups were performed using the DESeq2 R package (1.20.0) and resulting *p*-values were adjusted using the Benjamini and Hochberg’s approach for controlling the false discovery rate. Genes with an adjusted the value of *p*<0.05 found by DESeq2 were assigned as differentially expressed. ClusterProfiler R package was used to test the statistical enrichment of DEGs in Kyoto encyclopedia of genes and genomes (KEGG) pathways.

### Real-Time Quantitative PCR and Semi-Quantitative RT-PCR

For RT-qPCR, RNA was reverse transcribed into cDNA using HiScript III RT SuperMix for qPCR (+g DNA wiper; Vazyme Biotech. Co., Ltd., Cat No./ID: R323-01, Nanjing, China), and the cDNA was amplified using ChamQ Universal SYBR qPCR Master Mix (Vazyme Biotech. Co., Ltd., Cat No./ID: Q711, Nanjing, China) on the LightCycler® 96 Instrument (F. Hoffmann-La Roche Ltd., Switzerland). All the primers used for RT-qPCR can be found in Supplementary Table S1. The amplification reactions were performed in a total volume of 10μl, containing 5μl 2×ChamQ SYBR qPCR mix, 0.6μl forward and reverse primers (10μM), 1μl cDNA (10 times diluted), and 3.4μl ddH_2_O. The RT-qPCR amplification program was as follows: 95°C for 10min; 95°C for 10s, 60°C for 30s, and amplification for 40cycles. Each sample comprised three technical replications. Analysis of the relative gene expression data was conducted using the 2^−ΔC’t^ (Livak and Schmittgen, 2001). For semi-quantitative RT-PCR, RNA was reverse transcribed into cDNA using iScript™ Reverse Transcription Supermix for RT-qPCR (BIO-RAD, Cat No./ID: 6031, CA, United States), and cDNA was amplified using DreamTaq DNA Polymerase (Thermo Fisher Scientific, Cat No./ID: EP0702, MA, United States). The amplification reactions were performed in a total volume of 20μl, containing 0.1μl DreamTaq DNA polymerase, 2μl 10×DreamTaq buffer, 0.4μl dNTP mixture (5mM each), 1μl forward and reverse primers (10μM), 2μl cDNA, and 14.5μl ddH_2_O. The semi-quantitative RT-PCR amplification program was as follows: 95°C for 5min; 95°C for 30s, 52°C for 30s, and 72°C for 30s (30cycles); and 72°C for 30s.

### Accession Numbers

Sequence data from this article can be found under the following accession numbers. For genes from tobacco and *O*. *sativa*, sequences can be found in the NCBI database^2^: *NtCBL5A* (XM_016642104.1), *NsylCBL5* (KM658159.1), *NtomCBL5* (XM_018767788.1), *OsCBL1* (DQ201195), *OsCBL2* (DQ201196), *OsCBL3* (DQ201197), *OsCBL4* (DQ201198), *OsCBL5* (DQ201199), *OsCBL6* (DQ201200), *OsCBL7* (DQ201201), *OsCBL8* (DQ201202), *OsCBL9* (DQ201203), and *OsCBL10* (DQ201204). For genes from *S*. *lycopersicum*, sequences can be found in the SGN database^3^: *SlCBL1* (Solyc06g060980), *SlCBL2* (Solyc12g015870), *SlCBL4-1* (Solyc08g036590), *SlCBL4-2* (Solyc12g055920), *SlCBL8* (Solyc08g054570), and *SlCBL10* (Solyc08g065330). For genes from *A*. *thaliana*, sequences can be found in the TAIR database^4^: *AtCBL1* (AT4G17615), *AtCBL2* (AT5G55990), *AtCBL3* (AT4G26570), *AtCBL4* (AT5G24270), *AtCBL5* (AT4G01420), *AtCBL6* (AT4G16350), *AtCBL7* (AT4G26560), *AtCBL8* (AT1G64480), *AtCBL9* (AT5G47100), and *AtCBL10* (At4G33000). The RNA-seq data from this article can be found in the National Center for Biotechnology Information Gene Expression Omnibus (GEO) data repository under accession number GSE181164.^5^

### Statistical Analysis

Statistical analysis was done using IBM SPSS Statistics 23 software. Significant differences were examined by one-way ANOVA using the LSD test at *p*<0.05 and *p*<0.001. The figures were drawn by GraphPad Prism 6.0.

## Results

### The Cloning and Expression Analysis of *NtCBL5A*

*Nicotiana tabacum* is a natural allotetraploid derived from two diploid progenitors: *N. sylvestris* as the maternal genome donor and *Nicotiana tomentosiformis* as the paternal genome donor (Yukawa et al., 2006). We predicted and cloned 12 *NsylCBLs* based on the *N. sylvestris* genome data published in NCBI (see footnote 2; An et al., 2020). One of these 12 *NsylCBLs* (NCBI reference sequence: XM_009758979.1) had the closest phylogenetic relationship with *AtCBL5* (At4g01420) and therefore was named *NsylCBL5* (GenBank: KM658159.1). The ortholog of *NsylCBL5* in *N. tabacum* L. cv. Zhongyan 100 was subsequently cloned and named *NtCBL5A* (NCBI reference sequence number: XM_016642104.1). The coding sequence (CDS) of *NtCBL5A* is identical to the CDS of *NsylCBL5* and has 28 nucleotide differences with the CDS of *NtomCBL5* (NCBI reference sequence number: XM_018767788.1; Supplementary Figure S1A).

**Figure 1:**
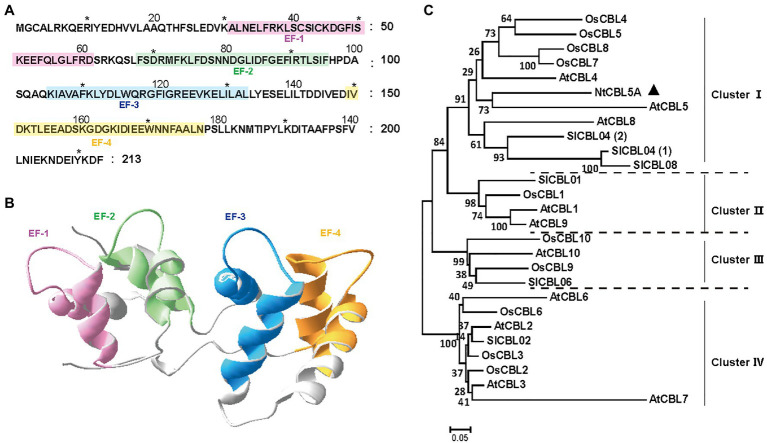
Structure and phylogenetic relationship of NtCBL5A. **(A)** Amino acid sequence of NtCBL5A and the positions of four EF-hands predicted by SMART. **(B)** The 3D structure of NtCBL5A predicted by SWISS-MODEL. **(C)** Phylogenetic analysis of NtCBL5A and all known CBL members from *Arabidopsis thaliana*, *Oryza sativa*, and *Solanum lycopersicum*. The amino acid sequences of AtCBLs, OsCBLs, and SlCBLs were downloaded from TAIR (https://www.arabidopsis.org/), NCBI (https://www.ncbi.nlm.nih.gov/), and Sol Genomics Network (https://solgenomics.net/), respectively. The phylogenetic tree was constructed by MEGA6 using the Neighbor-Joining method.

The CDS of *NtCBL5A* is 642bp in length, encoding a 213-amino-acid protein. The NtCBL5A protein is predicted to have four potential elongation factor hands (EF-hands) by the SMART^6^ (Letunic and Bork, 2018) and SWISS-MODEL^7^ (Waterhouse et al., 2018; Figures 1A,B). The EF-hand motif is the conserved domain of CBL proteins with an α-helix-loop-α-helix structure that binds Ca^2+^ (Sánchez-Barrena et al., 2013). A phylogenetic tree of NtCBL5A with all identified CBL proteins in *A. thaliana*, *O. sativa*, and *S. lycopersicum* distributed the CBL members over four clusters, and NtCBL5A was included in ClusterIwith closest phylogenetic relationship to AtCBL5 (Figure 1C).

**Figure 2:**
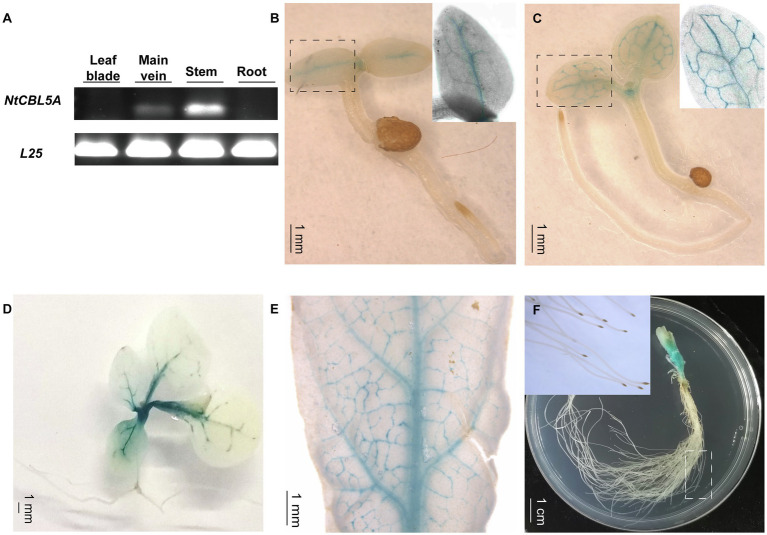
The expression profile of *NtCBL5A* in tobacco plants. **(A)** The expression profile detection in different tissues by semi-quantitative RT-PCR at 30days after germination (DAG), *L25* is the reference gene (Schmidt and Delaney, 2010). **(B-F)** The GUS staining of different tissues of *ProNtCBL5A::GUS* plants at different growth stages. They are seedlings at 3 DAG **(B)**, seedlings at 5 DAG **(C)**, seedlings at 20 DAG **(D)**, leaves of the seedlings at 40 DAG **(E)**, and stem and root tissues of the seedlings at 40 DAG **(F)**, respectively.

Semi-quantitative RT-PCR indicated that *NtCBL5A* is specifically expressed at a higher level in stems and a relatively lower level in main veins of young tobacco seedlings, and it is not detectable in roots and leaf blades (with main veins removed) at 30DAG (Figure 2A). We examined tissue-specific expression in more detail using independent *ProNtCBL5A::GUS* transgenic tobacco lines with a 2,780bp upstream regulatory region of *NtCBL5A* including the promoter (Supplementary Figure S1B) driving expression of a *GUS* reporter gene. Consistent with the semi-quantitative RT-PCR result, strong GUS activity was mainly detected in veins and stems of tobacco seedlings (Figures 2B–F). At 3 and 20 DAG, GUS staining was only observed in the veins and the top of the stem (Figures 2B–D). At 40 DAG, GUS staining was still limited to the veins and the stem (Figures 2E,F).

### Overexpression of *NtCBL5A* Induces Salt Supersensitivity With Necrotic Lesions on Leaves

**Figure 3:**
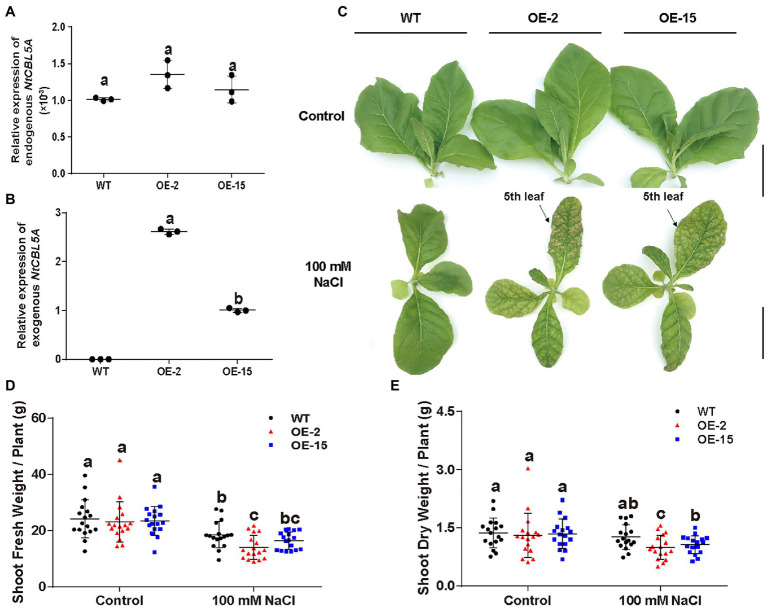
The detection of *NtCBL5A* expression and above-ground phenotype of wild-type (WT) and *NtCBL5A*-overexpressing (OE) lines (OE-2 and OE-15) under control conditions and salt stress (100mM NaCl). Scale bars=10cm. **(A)** Relative expression analysis of endogenous *NtCBL5A* (the *NtCBL5A* driven by 35S promoter) determined by RT-qPCR in different tissues of tobacco seedlings at 4days after the start of treatment (DAT). The expression of *NtCBL5A* is relative to the reference gene *L25* and the seedlings are 30-DAG old. **(B,C)** Relative expression analysis of endogenous *NtCBL5A* and exogenous *NtCBL5A* (the *NtCBL5A* driven by its own promoter in tobacco) determined by RT-qPCR in whole plants. The expression of *NtCBL5A* is relative to the reference gene *L25* and the seedlings are 12-DAG old. The reverse primer pCHF3-Allcheck-1 used for amplifying exogenous *NtCBL5A* was designed according to the sequence of the overexpression vector pCHF3, referring to pCHF3-Allcheck-2 (Shi et al., 2021). **(D)** The shoot phenotype of WT and *NtCBL5A*-OE lines at 9 DAT. **(E,F)** The shoot fresh weight and dry weight of WT and *NtCBL5A*-OE lines at 9 DAT. Error bars indicate ±SD (*n*=3 for gene expression detection, *n*=17 for shoot fresh/dry weight determination), different letters above bars (a, b, and c) indicate significant statistical difference based on one-way ANOVA with LSD test (*p*<0.05).

**Figure 4:**
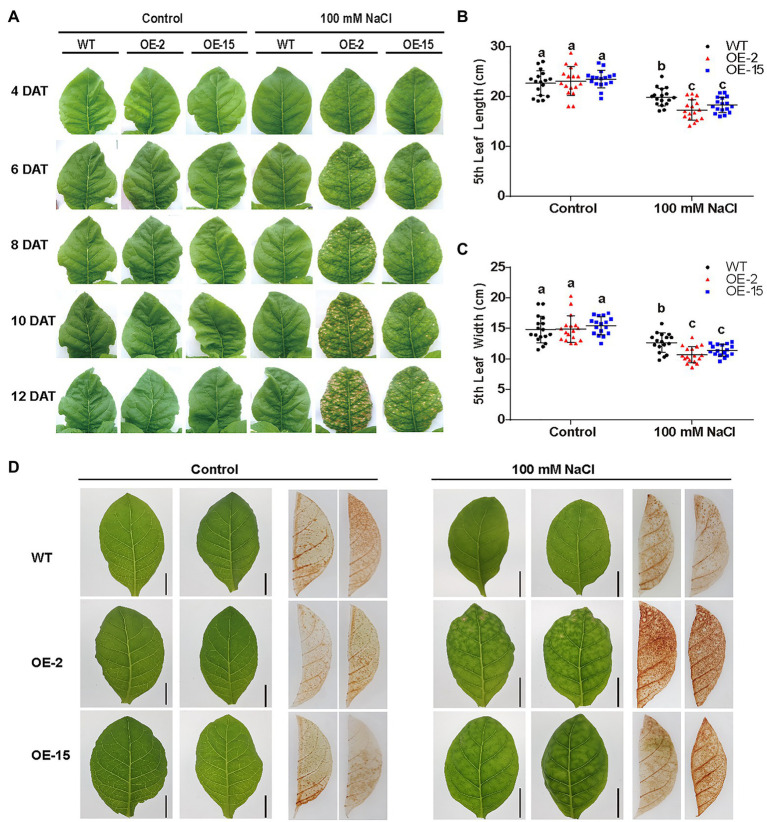
The determination of physiological parameters in wild-type (WT) and *NtCBL5A*-overexpressing lines (OE-2 and OE-15). **(A)** The phenotype of the fifth leaf of WT and *NtCBL5A*-OE lines under control conditions and salt stress (100mM NaCl) from 4 to 13 DAT. **(B,C)** Leaf length and leaf width determination of the fifth leaf at 8 DAT. **(D)** DAB staining of tobacco under control conditions and salt stress (100mM NaCl) at 6 DAT. Error bars indicate ±SD (*n*=17), different letters above bars (a, b, and c) indicate significant statistical difference based on one-way ANOVA with LSD test (*p*<0.05). Scale bars=2cm.

Two independent homozygous *NtCBL5A*-OE lines (OE-2, OE-15) with different overexpression levels were selected for salt tolerance evaluation (Figures 3A,B). Wild-type (WT) and *NtCBL5A*-OE lines were treated under control conditions (1/2 Hoagland’s nutrient solution) and saline conditions (1/2 Hoagland’s nutrient solution with 100mM NaCl) in a hydroponic growth system. Under control conditions, there were no phenotype differences between WT and *NtCBL5A*-OE lines. Under saline conditions, constitutive overexpression of *NtCBL5A* led to salt supersensitivity (Figure 3C). There were leaf chlorosis spots on *NtCBL5A*-OE leaves at the early stage of salt stress that developed fast into severe necrotic lesions within 2weeks (Figure 4A). In each *NtCBL5A*-OE plant, the fifth leaf that emerged just before the initiation of the salt treatment showed the most severe necrotic lesions (Figure 3C), and the occurrence of necrotic lesions started from leaf tip and leaf margin (Figure 4A). The overexpression level of *NtCBL5A* appeared to be related to the necrotic phenotype, for the OE-2 line with higher *NtCBL5A* expression level showed more severe necrotic lesions than OE-15 (Figure 4A).

Shoot dry weight and fresh weight of each tobacco line were reduced significantly under salt stress, and the reduction of *NtCBL5A*-OE lines was larger than that of WT at 9DAT (Figures 3D,E). Under saline conditions, the length and width of the fifth *NtCBL5A*-OE leaves were more reduced with curly and narrow leaf shapes (Figure 4). In addition, reactive oxygen species (ROS) accumulation in the fifth *NtCBL5A*-OE leaves was higher than that in WT leaves under salt stress at 2 and 6DAT (Supplementary Figure S2; Figure 4D). Root lengths of *NtCBL5A*-OE lines and WT were similarly affected by salinity, but root fresh weight was reduced more in the *NtCBL5A*-OE lines than in WT at 9DAT (Supplementary Figure S3).

### The Necrotic Lesions Are Specifically Induced by High Na^+^ in the Nutrient Solution

**Figure 5:**
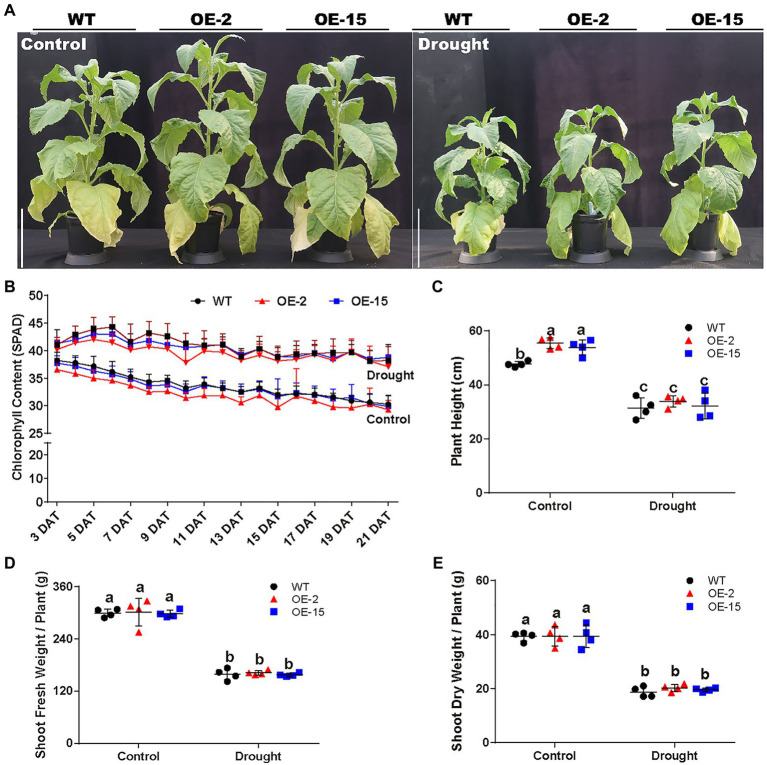
Phenotypic analysis of WT and *NtCBL5A*-OE lines (OE-2 and OE-15) under control conditions and drought stress. Scale bars=30cm. **(A)** The phenotype of WT and *NtCBL5A*-OE lines under control conditions and drought stress at 21DAT and 64DAG. **(B)** The chlorophyll content of the eigth leaf of WT and *NtCBL5A*-OE lines from 3 to 21 DAT. **(C–E)** The plant height, fresh shoot biomass, and dry shoot biomass of WT and *NtCBL5A*-OE lines at 21 DAT. Error bars indicate ±SD (*n*=4), different letters above bars (a, b, and c) indicate significant statistical difference based on one-way ANOVA with LSD test (*p*<0.05).

**Figure 6:**
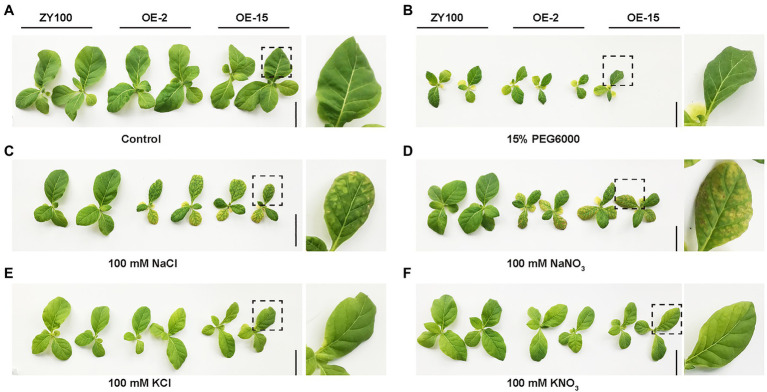
Ion and osmotic stress evaluation on WT and *NtCBL5A*-overexpressing lines (OE-2 and OE-15) at 9DAT. **(A)** The phenotype of WT and *NtCBL5A*-OE lines under control condition (1/2 Hoagland’s nutrient solution). **(B)** The phenotype of WT and *NtCBL5A*-OE lines under osmotic stress (1/2 Hoagland’s nutrient solution added 15% PEG6000). **(C–F)** The phenotype of WT and *NtCBL5A*-OE lines under ion stresses (1/2 Hoagland’s nutrient solutions added 100mM NaCl, 100mM NaNO_3_, 100mM KCl, and 100mM KNO_3_, respectively). Scale bars=10cm. Fourth leaves under light (Light), covered by aluminum-foil paper (Covered), and under dark (Dark), which were zoomed in at the right part of the panel.

The response to the osmotic component of salt stress bears similarity to the response to drought (Bartels and Sunkar, 2005). Therefore, the effect of drought stress on *NtCBL5A*-OE plants was determined as well. WT and *NtCBL5A*-OE lines were exposed to drought stress in pots under greenhouse conditions. At 21DAT, all the lines showed reduced growth compared to the control plants with no water limitation, but there were no significant phenotype differences between the WT and *NtCBL5A*-OE plants under drought stress (Figure 5A). Under drought stress, plant height, and fresh and dry shoot weight of each line were decreased, while chlorophyll content of each line was significantly increased compared to control conditions (Figures 5B–E). There were no differences between WT and *NtCBL5A*-OE lines under drought stress (Figures 5B–E); suggesting that osmotic stress alone did not trigger necrotic lesions on *NtCBL5A*-OE leaves. A 15% PEG6000 treatment was conducted to confirm that the necrotic lesions on transgenic lines are not caused by osmotic stress. Indeed, the PEG6000-induced osmotic stress did not induce leaf necrosis in OE-2 and OE-15 lines (Figure 6B).

Under saline conditions, both Na^+^ and Cl^−^ can be toxic to the plant (Munns and Tester, 2008). To identify the ion that is responsible for the necrotic phenotype of *NtCBL5A*-OE lines under salt stress, the plants were exposed not only to 100mM NaCl, but also to 100mM NaNO_3_, 100mM KNO_3_, and 100mM KCl (Quan et al., 2007). At 9DAT, both WT and *NtCBL5A*-OE lines exhibited reduced growth under all treatments relative to control conditions (Figure 6). *NtCBL5A*-OE lines showed leaf necrosis only under NaCl and NaNO_3_ treatments but not under KNO_3_ and KCl treatments (Figures 6C–F), suggesting that the necrotic lesions on *NtCBL5A*-OE lines are specifically induced by high levels of Na^+^ in the nutrient solution.

### Overexpression of *NtCBL5A* Enhances the Sensitivity of Transgenic Tobacco Leaves to Na^+^

**Figure 7:**
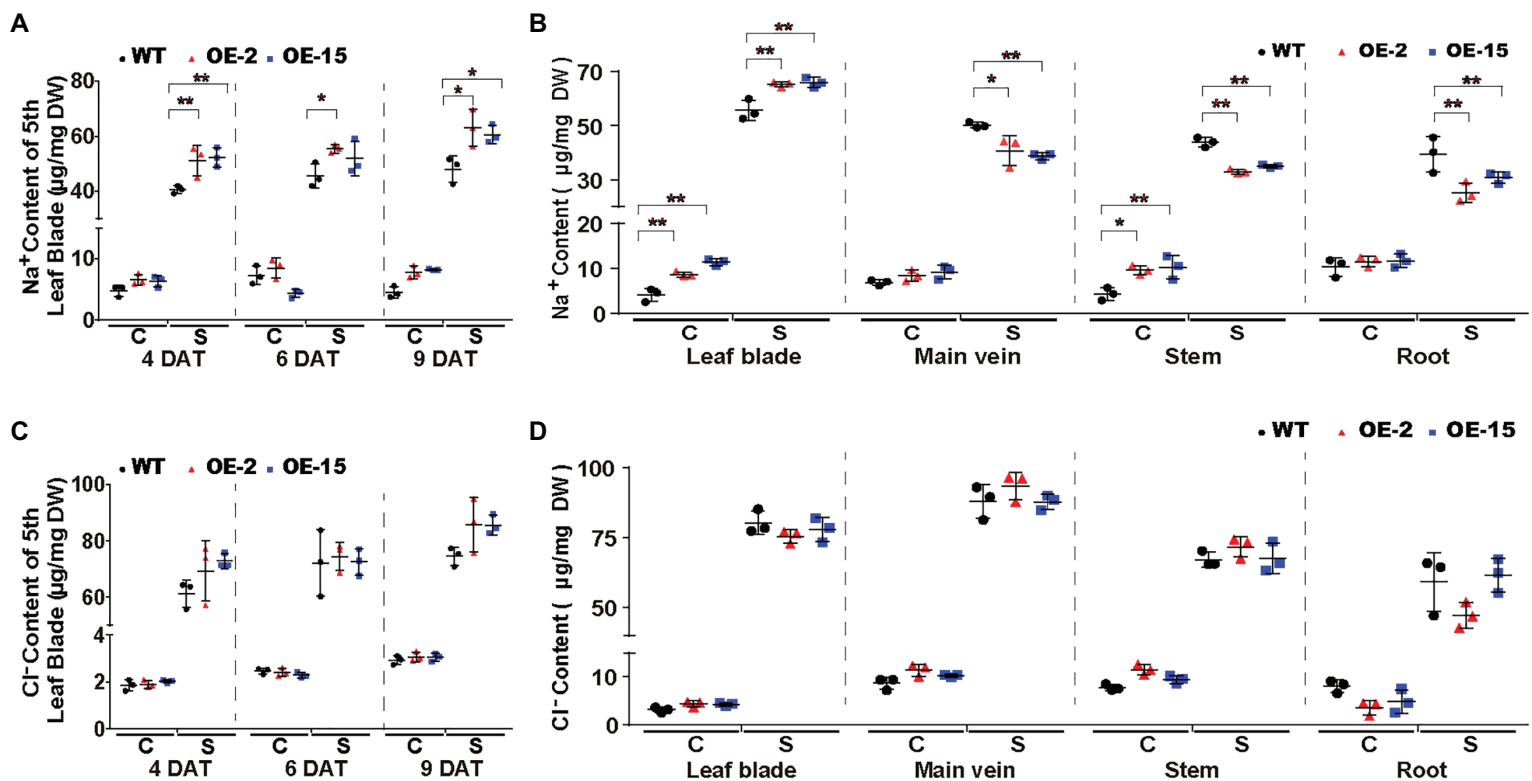
Na^+^ and Cl^−^ contents in WT and *NtCBL5A*-overexpressing lines (OE-2 and OE-15) under control conditions and salt stress (100mM NaCl). **(A,C)** Na^+^ and Cl^−^ contents in the fifth leaf blades (with main veins removed) at 4, 6, and 9 DAT. **(B,D)** Na^+^ and Cl^−^ contents in different tissues (leaf blades: all leaves with main veins removed; main veins: main veins from all leaves; stems; and roots) at 10 DAT. C means control conditions, while S means salt stress. Error bars indicate ±SD (*n*=3), every biological replication is a mixed pool of three plants. One-way ANOVA with LSD test (^*^p<0.05 and ^**^p<0.01) was used to analyze statistical significance.

To elucidate the cause of the salt-induced necrotic lesions in *NtCBL5A*-OE leaves, we measured the ion contents in the fifth leaf blades (with main veins removed) of all tobacco lines at three treatment time points (4, 6, and 9DAT). It needs to mention first that there was no significant difference between leaf water contents of WT and *NtCBL5A*-OE (Supplementary Figure S4A). Na^+^ contents in the fifth leaf blades of all lines were strongly increased under salt stress relative to control conditions and increased with treatment time. Compared to WT, Na^+^ contents in the fifth leaf blades of *NtCBL5A*-OE lines were higher under salt stress but the difference was not large (Figure 7A). More specifically, during the 4~9DAT, the Na^+^ contents in the 5th leaf blades of WT, OE-2, and OE-15 tobacco plants are in the range of 39.18~51.64, 44.95~69.71, and 47.36~64.31μg/mg, respectively. In other words, the 5th leaf blades of WT lines could reach a similar Na^+^ content level to that of OE lines with treatment time, but they did not exhibit any necrotic lesions at all during the treatment period (Figures 4A, 7A). Cl^−^ contents in the 5th leaf blades of all lines were strongly increased under salt stress relative to control conditions and increased with time, but there was no significant difference between WT and *NtCBL5A*-OE lines under salt stress (Figure 7C). K^+^, Ca^2+^, and Mg^2+^ contents in all lines were significantly decreased under salt stress but also for these ions there was still no significant difference between WT and *NtCBL5A*-OE plants (Supplementary Figures S4B–D).

We also measured the Na^+^ and Cl^−^ contents in different tissues (leaf blades: all leaves with main veins removed; main veins: main veins from all leaves; stems; and roots) of all lines under control conditions and 100mM NaCl stress at 10DAT. Under salt stress, Na^+^ contents in all tissues of all lines were strongly increased relative to control conditions, and Na^+^ contents in leaf blades of *NtCBL5A*-OE lines were significantly higher than those of WT (Figure 7B). In contrast, Na^+^ contents in main veins, stems, and roots of OE-2 and OE-15 were significantly lower than those of WT under salt stress (Figure 7B). Cl^−^ contents in all tissues of all lines were strongly increased under salt stress relative to control conditions but there was no significant difference between different lines (Figure 7D). Taken together, ion content data suggested that the overexpression of *NtCBL5A* may promote Na^+^ loading into leaf blades, but the necrotic lesions are caused by the increased Na^+^ sensitivity of *NtCBL5A*-OE leaves.

### Differentially Expressed Genes in *NtCBL5A*-OE Leaves Under Salt Stress Were Analyzed

**Figure 8:**
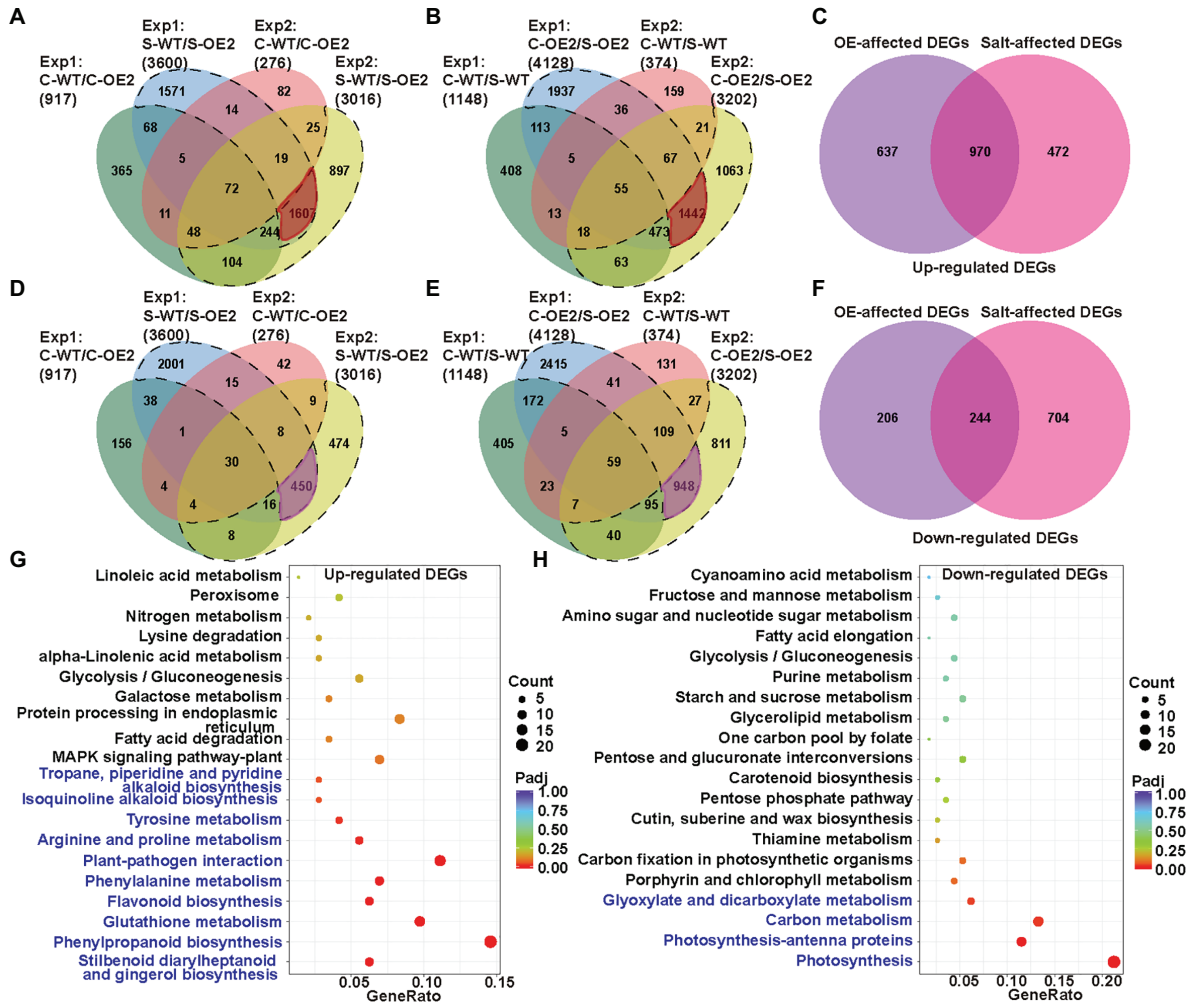
The analysis of leaf transcriptome data of WT and the *NtCBL5A*-overexpressing line (OE-2) at 4 DAT. **(A)** Venn diagram with four up-regulated gene sets: Experiment 1 (Exp1)-C-WT/C-OE2, Exp 1-S-WT/S-OE2, Exp 2-C-WT/C-OE2, and Exp 2-S-WT/S-OE2. **(B)** Venn diagram with four upregulated gene sets: Exp1-C-WT/S-WT, Exp 1-C-OE2/S-OE2, Exp 2-C-WT/S-WT, and Exp 2-C-OE2/S-OE2. **(C)** Venn diagram with two sets: OE-affected upregulated differentially expressed genes (DEGs) and Salt-affected upregulated DEGs. **(D)** Venn diagram with four downregulated gene sets: Exp 1-C-WT/C-OE2, Exp 1-S-WT/S-OE2, Exp 2-C-WT/C-OE2, and Exp 2-S-WT/S-OE2. **(E)** Venn diagram with four downregulated gene sets: Exp 1-C-WT/S-WT, Exp 1-C-OE2/S-OE2, Exp 2-C-WT/S-WT, and Exp 2-C-OE2/S-OE2. **(F)** Venn diagram with two sets: OE-affected down-regulated DEGs and Salt-affected downregulated DEGs. **(G,H)** Kyoto encyclopedia of genes and genomes (KEGG) enrichment of upregulated genes and downregulated genes. The pathways labeled in blue were significantly enriched pathways. Count: the number of DEGs, bigger circle means more DEGs number; GeneRatio: the number of DEGs/the total number of genes in this pathway; padj: *p*, padj<0.05 means significant difference, redder color means greater significance. The raw data of RNA-seq can be found in GEO data repository with the accession number GSE181164, in which samples were named as C-WT-1, S-WT-1, C-OE2-1, S-OE2-1, C-WT-2, S-WT-2, C-OE2-2, and S-OE2-2. “C” refers to “Control,” “S” refers to “Salt,” “WT” refers to “wild-type,” “OE2” refers to the OE2 line of *NtCBL5A*-overexpressing lines, “1” refers to “Experiment 1,” and “2” refers to “Experiment 2.”

To further identify the genes and pathways involved in the necrotic phenotype of *NtCBL5A*-OE lines, the leaf transcriptome profiling of WT and OE-2 lines grown under control conditions and salt stress (100mM NaCl) at 4DAT were sequenced and compared. Two datasets of DEGs were made in which we identified the genes that were differentially expressed as a result of the overexpression of *NtCBL5A*: Control-WT vs. Control-OE2 (C-WT/C-OE2) and Salt-WT vs. Salt-OE2 (S-WT/S-OE2). Another two datasets were also used to identify the transcripts that were responsive to the salt treatments: Control-WT vs. Salt-WT (C-WT/S-WT) and Control-OE2 vs. Salt-OE2 (C-OE2/S-OE2). DEGs from C-WT/C-OE2 and S-WT/S-OE2 were compared to select the transcripts affected by *NtCBL5A* overexpression only under salt stress (dotted lines in Figures 8A,D; Supplementary Figure S5). We also compared DEGs from C-WT/S-WT and C-OE2/S-OE2 to identify the specific transcripts affected by salt stress and only in *NtCBL5A*-OE lines (dotted lines in Figures 8B,E; Supplementary Figure S5). This procedure was done for two independent experiments, and only DEGs that were identified in both experiments were considered (highlighted part in Figures 8A,B,D,E). The OE-affected DEGs and salt-affected DEGs together resulted in 2079 upregulated DEGs and 1,154 down-regulated DEGs (Figures 8C,F), strongly affected by the combination of *NtCBL5A* overexpression and salt stress.

The upregulated DEGs were enriched in 10 KEGG (Kanehisa et al., 2020) pathways (padj<0.05; Figure 8G). Among them, “plant-pathogen interaction” (KEGG ID: sly04626) and “MAPK signaling pathway-plant” (KEGG ID: sly04016) attracted our attention because many DEGs identified as belonging to these two pathways are related to HR and cell death, including *PATHOGENESIS RELATED PROTEIN 1a* (*PR1a*), *PR1b*, *PR1c*, *PR-Q*, *PR-R* major form, *PR-R* minor form, *ETHYLENE RESPONSE FACTOR 1* (*ERF1*), *ENHANCED DISEASE SUSCEPTIBILITY 1* (*EDS1*, SA-related signal transducers), and *RPM1-INTERACTING PROTEIN 4* (*RIN4*). Besides, Ca^2+^ channels *CYCLIC NUCLEOTIDE-GATED ION CHANNEL* (*CNGC*) and other two types of Ca^2+^-sensor genes *CML* and *CDPK* were also enriched in the MAPK signaling pathway-plant. Interestingly, these genes are highly upregulated only under the combination of *NtCBL5A* overexpression and salt stress. The down-regulated DEGs were enriched in four KEGG pathways (padj<0.05; Figure 8H). In “photosynthesis” (KEGG ID: sly00195) and “photosynthesis-antenna proteins” (KEGG ID: sly00196) pathways, many genes related to photosystem I (e.g., *PsaD*, *PsaH*, and *PsaE*), photosystem II (e.g., *PsbD*, *PsbQ*, and *PsbW*), photosynthetic electron transport (*PetE*, *PetF*, and *PetH*), and light-harvesting chlorophyll protein complex (e.g., *Lhca1-5*, *Lhcb1*, and *Lhcb3-6*) were significantly downregulated.

### Plant Defense- and Cation Homeostasis-Related Genes Are Regulated in *NtCBL5A*-OE Leaves Under Salt Stress

**Figure 9:**
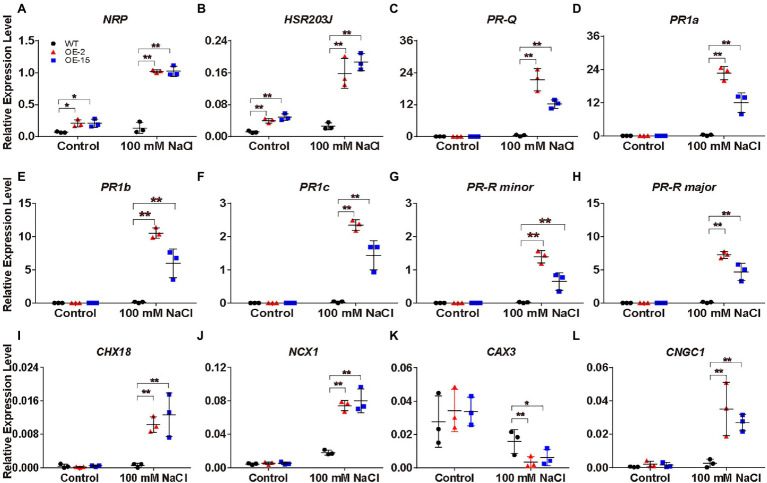
Relative expression analysis of plant defense-related marker genes, Na^+^ homeostasis- and Ca^2+^ homeostasis-related genes determined by RT-qPCR in tobacco leaves. (A-L) The expression of these genes is relative to the reference gene *L25* under control conditions and salt stress (100mM NaCl) at 4days after the start of treatment. Their gene IDs in the reference tobacco genome database (ftp://ftp.solgenomics.net/genomes/Nicotiana_tabacum/edwards_et_al_2017/assembly/Nitab-v4.5_genome_Chr_Edwards2017.fasta.gz) are *N-RICH PROTEIN* (*NRP*; Nitab4.5_0000798g0120), *HSR203J* (Nitab4.5_0002719g0120), *PR-Q* (Nitab4.5_0003207g0080), *PR1a* (Nitab4.5_0003771g0010), *PR1b* (Nitab4.5_0005400g0020), *PR1c* (Nitab4.5_0004861g0040), *PR-R* minor (Nitab4.5_0004097g0050), *PR-R* major (Nitab4.5_0000360g0100), *Cation/H^+^ EXCHANGER 18* (*CHX18*; Nitab4.5_0006998g0030), *Na^+^/Ca^2+^ EXCHANGER 1* (*NCX1*; Nitab4.5_0005404g0030), *CATION/PROTON 3* (*CAX3*; Nitab4.5_0000102g0080), and *CNGC1* (Nitab4.5_0000258g0120). Error bars indicate ±SD (*n*=3). One-way ANOVA with LSD test (^*^p<0.05 and ^**^p<0.01) was used to analyze statistical significance.

Under salt stress, *NtCBL5A-*OE leaves exhibited necrotic lesions that bear resemblance to hypersensitive reaction (HR)-like cell death in plant response to pathogen infection. To understand the causes of the necrotic lesions, we specifically examined the expression of the HR marker genes *N-RICH PROTEIN* (*NRP*; Ludwig and Tenhaken, 2001) and *HYPERSENSITIVE-RELATED 203J* (*hypersensitive-related 203J*; Pontler et al., 1994), as well as the plant defense-related genes in the “Plant-pathogen interaction” pathway and “MAPK signaling pathway-plant” pathway (Figure 8G). *NPR* and *HSR203J* genes were strongly upregulated in *NtCBL5A*-OE leaves under salt stress (Figures 9A,B). In addition, the expression levels of plant defense-related genes like *PR* genes (*PR1a*, *PR1b*, *PR1c*, *PR-Q*, and *PR-R*; Sinha et al., 2014), *EDS1* (Lapin et al., 2020), *RIN4* (Ray et al., 2019), *ERF1* (Lorenzo et al., 2003), and *CATALASE 1* (*CAT1*; Tran and Jung, 2020) were much higher in *NtCBL5A*-OE leaves than those in WT leaves under salt stress (Figures 9C–H; Supplementary Figures S6A–D). Taken together, these data suggested that salinity stress activates an immunity-related response specifically in the *NtCBL5A*-OE lines.

Under saline conditions, Na^+^ accumulation in *NtCBL5A*-OE leaves was slightly higher than that in WT leaves (Figure 7A). Therefore, we hypothesized that *NtCBL5A* overexpression may affect the expression of several cation homeostasis-related genes involved in salt stress response. Based on the transcriptome analysis, several genes required for K^+^, Na^+^, and Ca^2+^ homeostasis were significantly up or downregulated by combined condition of *NtCBL5A* overexpression and salt stress. The expression profile of these genes was validated by RT-qPCR. *Cation/H^+^ EXCHANGER 18* (*CHX18*) and *Na^+^/Ca^2+^ EXCHANGER 1* (*NCX1*) gene were strongly upregulated (Figures 9I,J), while *vacuolar CATION/PROTON 3* (*CAX3*) gene involved in ion vacuolar compartmentalization (Martinoia et al., 2007; Zhao et al., 2007) was downregulated in WT and even more strongly inhibited in *NtCBL5A*-OE lines under saline conditions (Figure 9K). In addition, the expression of Ca^2+^ channels *CNGC1* was upregulated in *NtCBL5A*-OE leaves under saline conditions (Figure 9L). The results of RT-qPCR were consistent with the transcriptome data and indicated that *NtCBL5A* overexpression greatly affects the expression of cation homeostasis-related genes under salt stress.

### Photosynthetic Machinery-Related Genes Are Strongly Inhibited in *NtCBL5A*-OE Leaves Under Salt Stress

**Figure 10:**
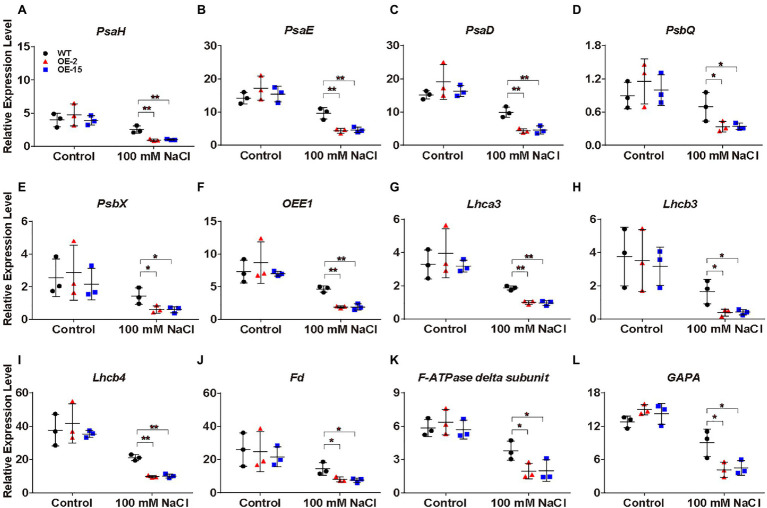
Relative expression analysis of photosynthesis-related genes determined by RT-qPCR in tobacco leaves. (A-L) The expression of these genes is relative to the reference gene *L25* under control conditions and salt stress (100mM NaCl) at 4DAT. Their gene IDs in the reference tobacco genome database (ftp://ftp.solgenomics.net/genomes/Nicotiana_tabacum/edwards_et_al_2017/assembly/Nitab-v4.5_genome_Chr_Edwards2017.fasta.gz) are *PsaH* (Nitab4.5_0000351g0060), *PsaE* (Nitab4.5_0000385g0230), *PsaD* (Nitab4.5_0014875g0010), *PsbQ* (Nitab4.5_0002345g0070), *PsbX* (Nitab4.5_0000073g0060), *OXYGEN EVOLVING ENHANCER PROTEIN 1* (*OEE1*; Nitab4.5_0000108g0110), *LIGHT-HARVESTING CHLOROPHYLL PROTEIN COMPLEX* (*Lhca3*; Nitab4.5_0000923g0200), *Lhcb3* (Nitab4.5_0012832g0010), *Lhcb4* (Nitab4.5_0011597g0020), *Fd* (Nitab4.5_0004129g0010), *F-ATPase delta subunit* (Nitab4.5_0006745g0030), and *GLYCERALDEHYDE-3-PHOSPHATE DEHYDROGENASE* A (*GAPA*; Nitab4.5_0010299g0040). Error bars indicate ±SD (*n*=3). One-way ANOVA with LSD test (^*^p<0.05 and ^**^p<0.01) was used to analyze statistical significance.

Under salt stress, *NtCBL5A*-OE leaves exhibited chlorotic spots developing into necrotic lesions from 4DAT (Figure 4A). To gain further insight into the causes of the chlorotic spots, we examined the expression levels of the photosynthesis essential genes in the “Photosynthesis” pathway and “Photosynthesis-antenna proteins” pathway (Figure 8H). *PsaH*, *PsaE*, and *PsaD* in photosystem I; *PsbQ*, *PsbX*, and *OXYGEN EVOLVING ENHANCER PROTEIN 1* (*OEE1*) in photosystem II; *LIGHT-HARVESTING CHLOROPHYLL PROTEIN COMPLEX* (*Lhca3*, *Lhcb3*, and *Lhcb4*); *FERREDOXIN* (*Fd*) in photosynthetic electron transport; *F-ATPase delta subunit*; and *GLYCERALDEHYDE-3-PHOSPHATE DEHYDROGENASE A* (*GAPA*) in Calvin cycle were examined, RT-qPCR results showed that their expression in both WT and *NtCBL5A*-OE leaves were significantly inhibited by salinity at 4 DAT (Figure 10). More importantly, their expression levels in *NtCBL5A*-OE leaves were significantly lower than that in WT leaves under salt stress (Figure 10). These data suggested that the photosynthetic machinery might be more severely affected in *NtCBL5A*-OE leaves than in WT leaves under salt stress at 4DAT.

## Discussion

The mechanisms of salt stress response have been the subject of many research studies. A lot of candidate genes involved in salt stress response have been identified, including genes responsible for sensing and signaling in roots, photosynthesis, Na^+^ accumulation in shoots and vacuoles, and accumulation of organic solutes (Munns and Tester, 2008). However, actual improvements to salt tolerance in crops have been limited, partially because salt tolerance is a polygenic trait controlled by quantitative loci (Ismail and Horie, 2017). Therefore, more pathway components in the salt stress response need to be explored. The strategy of generating a phenotype by targeted overexpression can be used as a powerful tool to identify the potential response pathway components (Prelich, 2012). In this study, constitutive overexpression of *NtCBL5A* greatly interferes with the normal salt stress response of tobacco and induces Na^+^-dependent salt supersensitivity with necrotic lesions on leaves. Analysis of this phenotype generated by *NtCBL5A* overexpression may provide insight into genes and relatively poorly explored pathways that are involved in the salt stress response of plants.

CBL-CIPK complexes act as important nodes in the plant signaling cascade, linking environmental stimuli with multiple biochemical and physiological responses. [Ca^2+^]_cyt_ triggered by salinity stress can be sensed by CBLs (Perochon et al., 2011). One CBL can interact with different CIPKs and each CIPK may phosphorylate diverse targets (Ma et al., 2020). Therefore, constitutive overexpression of a *CBL* gene may generate a cascade effect, overreacting to the [Ca^2+^]_cyt_ transients triggered by salinity stress and leading to unexpected phenotypes. To elucidate the mechanisms underlying the necrotic phenotype, the *NtCBL5A*-OE lines were evaluated at the physiological, biochemical, and molecular levels. We found that the necrotic phenotype was uniquely induced by high levels of Na^+^ rather than Cl^−^ and osmotic stress in the nutrient solution. Many genes related to cation homeostasis, plant immunity, and the photosynthetic machinery were affected at the transcriptional level in *NtCBL5A*-OE leaves under salt stress. The constitutive overexpression of *NtCBL5A* may make more potential pathway components in salt stress response detectable.

### Constitutive Overexpression of *NtCBL5A* May Interfere With the Network Responsible for Tobacco Tolerance

Under saline conditions, plants suffer from Na^+^ toxicity due to Na^+^ accumulation in the leaves (Munns and Tester, 2008). Generally, Na^+^ can accumulate to toxic concentrations earlier in old leaves than in younger growing leaves because the old leaves no longer expand and so no longer dilute the salt (Munns and Tester, 2008). In addition, basal zones of leaf blades accumulate more ions than tip zones because the dehydration process starts at the leaf tip (de Lacerda et al., 2003). Inconsistent with the normal pattern of Na^+^ accumulation in plants, however, the fifth leaf of *NtCBL5A*-OE tobacco developed the earliest and most severe necrotic lesions in this study (Figure 3C) and the occurrence of necrotic lesions started from leaf tip and leaf margin (Figure 4A). Ion content determination provides us with more information about the necrotic lesions. Although the severe necrotic lesions occurring on *NtCBL5A*-OE leaves were Na^+^-dependent, their total Na^+^ accumulation was only slightly higher than that of WT leaves. The difference in Na^+^ concentrations is statistically significant but not large. Specifically, during 4~9 DAT, the Na^+^ contents in the fifth leaves of WT, OE-2, and OE-15 are in the range of 39.18~51.64, 44.95~69.71, and 47.36~64.31μg/mg, respectively (Figure 7A). The 5th leaves of WT plants remained green with Na^+^ accumulating up to 51.64μg/mg, while *NtCBL5A*-OE lines exhibited obvious chlorotic spots or necrotic lesions with a similar level of Na^+^ accumulation in their leaves (Figures 4A, 7A). Taken the phenotypic analysis and Na^+^ determination together, it is possible that the distribution of Na^+^ over different tissues and cell organelles was affected by *NtCBL5A* overexpression. We did find the expression of several cation homeostasis-related genes, such as C*HX18*, *NCX1*, and vacuolar *CAX3* were regulated in salt-stressed *NtCBL5A*-OE leaves (Figure 9). To sum up, the compromised Na^+^-handling ability and Na^+^ homeostasis may contribute more to the formation of necrotic lesions on *NtCBL5A*-OE leaves.

Overexpression phenotypes often result from competition-based mechanisms (Prelich, 2012). The necrotic phenotype may result from the interference of ectopically expressed *NtCBL5A* with other components of the CBL-CIPK network that are important in the salt stress response of tobacco. CBL members with close phylogenetic relationships were shown to be able to interact with the same CIPK. Both AtCBL1 and AtCBL9 interact with AtCIPK23 to regulate K^+^ uptake under low-potassium conditions by activating K^+^ transporters (Li et al., 2006; Aleman et al., 2011; Ragel et al., 2015). In addition, AtCBL2 and AtCBL3 both interact with AtCIPK12 as crucial regulators of vacuole dynamics (Steinhorst et al., 2015), and work with AtCIPK3/9/23/26 at the tonoplast to sequester Mg^2+^ into vacuole to avoid high Mg^2+^ toxicity (Tang et al., 2015). Some CBL family members are supposedly able to compete for the same CIPK member, but they are “spatially isolated” because of different subcellular localization or tissue-specific expression. For example, both AtCBL4 and AtCBL10 interact with AtCIPK24 to regulate Na^+^ homeostasis under salt stress, but the AtCBL4-AtCIPK24 complex mainly works in roots for Na^+^ exclusion, the AtCBL10-AtCIPK24 complex mainly works in shoots for Na^+^ efflux or compartmentalization (Kim et al., 2007; Lin et al., 2009). This cooperation or competition of CBL family members suggests that ectopically expressed *NtCBL5A* might compete with other NtCBLs for the same NtCIPK and interfere with their functions, for instance with the NtCBL-NtCIPK complex that is regulating Na^+^ vacuolar compartmentalization and thus affect Na^+^ sequestration into the vacuole. As a result, Na^+^ might be less efficiently compartmentalized in vacuole, increasing the toxicity of Na^+^ in the cytoplasm. This would be in agreement with our hypothesis that the Na^+^ sensitivity of the *NtCBL5A*-OE lines under saline conditions might be partly caused by compromised distribution of Na^+^ within the leaves. This hypothesis can be verified by co-overexpression of *NtCBL5A* and its competing *NtCBL*, which may reverse the necrotic phenotype of *NtCBL5A*-OE leaves (Prelich, 2012).

### The Na^+^ Sensitivity of *NtCBL5A*-OE Leaves May Be Related to Defective Photosystems and ROS

Both transcriptome analyses and RT-qPCR results indicated that the expression of photosynthesis-related genes in *NtCBL5A*-OE leaves were significantly inhibited by salinity at 4 DAT (Figures 8H, 10), which is consistent with the dotted chlorosis phenotype at this time point (Figure 4A). To understand whether photosynthesis dysfunction is related to the salt-induced necrotic phenotype of *NtCBL5A*-OE leaves, we examined the expression levels of photosynthesis-related genes at very early stages of salt treatment (1 and 2DAT) when there were no chlorotic spots or necrotic lesions in *NtCBL5A*-OE leaves. RT-qPCR results showed that their expression levels in *NtCBL5A*-OE leaves were lower than those in WT already at 1DAT (Supplementary Figures S6E–P), which suggests that the photosynthetic machinery of *NtCBL5A*-OE leaves may be affected shortly after the start of salt treatment.

It has been reported that some leaf lesion-mimic phenotypes are connected to defective photosystems (Zulfugarov et al., 2014; Bruggeman et al., 2015; Wang et al., 2015; Tang et al., 2020). Light energy input exceeds energy utilization when CO_2_ assimilation and NADP^+^ regeneration in the Calvin cycle are inhibited by salinity-induced stomatal limitation, leading to overreduction of the electron transport chain and the generation of ROS (Attia et al., 2009; Hajiboland, 2014). If the capacity of the ROS scavenging system is not sufficient, excessive ROS will accumulate and lead to damage. The ROS accumulation in *NtCBL5A*-OE leaves was higher than that in WT under salt stress already at 2 and 6DAT (Supplementary Figure S2; Figure 4D). Possibly, the light energy input in *NtCBL5A*-OE leaves exceeds energy utilization when the Calvin cycle is more inhibited than light reaction in the photosynthesis of *NtCBL5A*-OE leaves under salt stress, and the resulting ROS generation in *NtCBL5A*-OE leaves might beyond the ROS scavenging ability and lead to the necrotic lesions.

The question then remains that how overexpression of *NtCBL5A* in combination with high Na^+^ in the root environment triggers excess ROS production and affects the photosynthetic machinery. It is remarkable that the expression of photosynthetic machinery-related genes already changed at 1 DAT, ROS appeared to be already elevated at 2 DAT, and chlorotic symptoms developed quickly after the start of salt treatment. This might indicate that the salt supersensitivity is initiated already during the early salinity response of the plant. Although it is generally accepted that the first response to salt stress in plants is triggered by the osmotic stress component of salinity, recent studies indicated that plants also specifically sense the presence of high Na^+^ in the soil at the early stages of salt stress (Lamers et al., 2020; Van Zelm et al., 2020). This triggers Ca^2+^ waves in the roots that even reach the leaves (Choi et al., 2014). Additionally, ROS are rapidly activated (Miller et al., 2010). Members of the CBL-CIPK signaling network may play a role in the translation of the second messenger Ca^2+^ (Manishankar et al., 2018), and the AtCBL1/9-AtCIPK26 complex was shown to be able to interact with AtRbohF, which is a member of the ROS burst-regulating Rboh gene family (Drerup et al., 2013). It is therefore conceivable that ectopic and constitutive overexpression of *NtCBL5A* may interfere with the Ca^2+^ and ROS-mediated response of plants following early sensing of high Na^+^ levels in the root environment. Further exploration of the reason for necrotic lesions on *NtCBL5A*-OE leaves might help to gain insight into this early Na^+^ response of plants as part of the response to salinity, and the crosstalk between the salt stress response and photosynthesis.

### The Na^+^ Sensitivity of *NtCBL5A*-OE Leaves May Be Related to Plant Immune Response

Transcriptome analysis provided additional information on cause of the fast-developing necrotic symptoms of *NtCBL5A*-OE tobacco. The expression of cell death- and immune response-related genes was induced in *NtCBL5A*-OE leaves under salt stress. These included *NRP* and *HSR203J*, which are immunity-related cell death HR marker genes that are activated as part of the plant disease defense and execution of the cell death program (Ludwig and Tenhaken, 2001). In addition, many plant defense-related genes like *PR genes*, *ERF1*, *EDS1*, and *RIN4* were also specifically strongly upregulated in *NtCBL5A*-OE lines under saline conditions (Figures 9A–H; Supplementary Figures S6A–D), suggesting that PAMP- and effector-triggered responses are induced in *NtCBL5A*-OE leaves under salt stress. The CBL-CIPK network has been widely reported to be involved in HR-related plant immunity to pathogens, such as SlCBL10-SlCIPK6 (Gutiérrez-Beltrán et al., 2017), AtCIPK6 (Sardar et al., 2017), OsCIPK15 (Kurusu et al., 2010), CaCIPK1 (Ma et al., 2019), TaCBL4-TaCIPK5 (Liu et al., 2018), TaCIPK10 (Liu et al., 2019), and MeCBL1/9-MeCIPK23 (Yan et al., 2018). Moreover, Cassava MeCBL1/9-MeCIPK23 positively regulates plant’s defense against *Xanthomonas axonopodis* pv. *manihotis via* affecting the expression of defense-related genes including *PR1*, *PR2*, *PR5*, and *NPR1* (nonexpresser of PR genes 1; Yan et al., 2018). Crosstalk between the response to abiotic and biotic stress and the role of plant immune-related genes in this crosstalk has been shown by others (Nejat and Mantri, 2017; Saijo and Loo, 2020), and it is possible that some plant immune-related DEGs in *NtCBL5A*-OE leaves under salt stress might be involved in the combined salt stress and biotic stress response.

## Data Availability Statement

The original contributions presented in the study are publicly available. RNA-seq data can be found here: National Center for Biotechnology Information Gene Expression Omnibus (GEO) data repository under accession number GSE181164.

## Author Contributions

JM conceived the original research plans, performed the experiments, and analyzed the data. HL, QW, and CL co-supervised the experiments. LA generated *ProNtCBL5A::GUS* transgenic tobacco lines. JY and ZM provided technical assistance to JM. SS generated *NtCBL5A*-OE lines in T0 generation. JM conceived the project and wrote the article with contributions of all other authors. YS and GL helped with manuscript revision. RV, YB, HL, QW, and CL supervised and completed the manuscript writing. HL and QW agreed to serve as the authors responsible for contact and ensure communication. All authors contributed to the article and approved the submitted version.

## Funding

This work was supported by the Fundamental Research Funds for China Agricultural Academy of Sciences (1610232021002), the Agricultural Science and Technology Innovation Program (ASTIP-TRIC02 and ASTIP-TRIC03), China Scholarships Council (CSC No. 201803250083), International Foundation of Tobacco Research Institute of CAAS (IFT202102), and International Exchange Scholarship of the GSCAAS.

## Conflict of Interest

The authors declare that the research was conducted in the absence of any commercial or financial relationships that could be construed as a potential conflict of interest.

## Publisher’s Note

All claims expressed in this article are solely those of the authors and do not necessarily represent those of their affiliated organizations, or those of the publisher, the editors and the reviewers. Any product that may be evaluated in this article, or claim that may be made by its manufacturer, is not guaranteed or endorsed by the publisher.

## Footnotes

^1^ ftp://ftp.solgenomics.net/genomes/Nicotiana_tabacum/edwards_et_al_2017/assembly/Nitab-v4.5_genome_Chr_Edwards2017.fasta.gz

^2^ https://www.ncbi.nlm.nih.gov/

^3^ https://solgenomics.net/

^4^ https://www.arabidopsis.org/

^5^ https://www.ncbi.nlm.nih.gov/geo/subs/?view=samples&series=181164

^6^ http://smart.embl-heidelberg.de/

^7^ https://swissmodel.expasy.org/

## References

[ref1] Aleman F. Nieves-Cordones M. Martinez V. Rubio F. (2011). Root K^+^ acquisition in plants: the *Arabidopsis thaliana* model. Plant Cell Physiol. 52, 1603–1612. doi: 10.1093/pcp/pcr096, PMID: 21771865

[ref2] An L. L. Mao J. J. Che H. Y. Shi S. J. Dong L. H. Xu D. Z.. (2020). Screening and identification of NsylCBL family members interacting with protein kinase NsylCIPK24a in *Nicotiana Sylvestris*. Mol. Plant Breed. 11, 1–10. doi: 10.5376/pgt.2020.10.0005

[ref3] Attia H. Karray N. Lachaâl M. (2009). Light interacts with salt stress in regulating superoxide dismutase gene expression in *Arabidopsis*. Plant Sci. 177, 161–167. doi: 10.1016/j.plantsci.2009.05.002

[ref4] Bartels D. Sunkar R. (2005). Drought and salt tolerance in plants. CRC Crit. Rev. Plant Sci. 24, 23–58. doi: 10.1080/07352680590910410

[ref5] Bruggeman Q. Raynaud C. Benhamed M. Delarue M. (2015). To die or not to die? Lessons from lesion mimic mutants. Front. Plant Sci. 6:24. doi: 10.3389/fpls.2015.00024, PMID: 25688254 PMC4311611

[ref6] Chakraborty K. Sairam R. K. Bhattacharya R. C. (2012). Differential expression of salt overly sensitive pathway genes determines salinity stress tolerance in *Brassica* genotypes. Plant Physiol. Biochem. 51, 90–101. doi: 10.1016/j.plaphy.2011.10.001, PMID: 22153244

[ref7] Chen L. Ren F. Zhou L. Wang Q. Q. Zhong H. Li X. B. (2012). The *Brassica napus* Calcineurin B-like 1/CBL-interacting protein kinase 6 (CBL1/CIPK6) component is involved in the plant response to abiotic stress and ABA signalling. J. Exp. Bot. 63, 6211–6222. doi: 10.1093/jxb/ers273, PMID: 23105131 PMC3481211

[ref8] Cheong Y. H. (2003). CBL1, a calcium sensor that differentially regulates salt, drought, and cold responses in *Arabidopsis*. Plant Cell 15, 1833–1845. doi: 10.1105/tpc.012393, PMID: 12897256 PMC167173

[ref9] Cheong Y. H. Sung S. J. Kim B. G. Pandey G. K. Cho J. S. Kim K. N.. (2010). Constitutive overexpression of the calcium sensor CBL5 confers osmotic or drought stress tolerance in *Arabidopsis*. Mol. Cell 29, 159–165. doi: 10.1007/s10059-010-0025-z, PMID: 20077023

[ref10] Cho J. H. Choi M. N. Yoon K. H. Kim K. N. (2018). Ectopic expression of *SjCBL1*, calcineurin B-like 1 gene from *Sedirea japonica*, rescues the salt and osmotic stress hypersensitivity in *Arabidopsis cbl1* mutant. Front. Plant Sci. 9:1188. doi: 10.3389/fpls.2018.01188, PMID: 30210512 PMC6123687

[ref11] Choi W. G. Toyota M. Kim S. H. Hilleary R. Gilroy S. (2014). Salt stress-induced Ca^2+^ waves are associated with rapid, long-distance root-to-shoot signaling in plants. Proc. Nati. Acad. Sci. U. S. A. 111, 6497–6502. doi: 10.1073/pnas.1319955111, PMID: 24706854 PMC4035928

[ref12] de Lacerda C. F. Cambraia J. Oliva M. A. Ruiz H. A. Prisco J. T. N. (2003). Solute accumulation and distribution during shoot and leaf development in two sorghum genotypes under salt stress. Environ. Exp. Bot. 49, 107–120. doi: 10.1016/S0098-8472(02)00064-3

[ref13] Dong L. H. Wang Q. Manik S. M. N. Song Y. F. Shi S. J. Su Y. L.. (2015). *Nicotiana sylvestris* calcineurin B-like protein NsylCBL10 enhances salt tolerance in transgenic *Arabidopsis*. Plant Cell Rep. 34, 2053–2063. doi: 10.1007/s00299-015-1851-4, PMID: 26318216

[ref14] Drerup M. M. Schlucking K. Hashimoto K. Manishankar P. Steinhorst L. Kuchitsu K.. (2013). The calcineurin B-like calcium sensors CBL1 and CBL9 together with their interacting protein kinase CIPK26 regulate the *Arabidopsis* NADPH oxidase RBOHF. Mol. Plant 6, 559–569. doi: 10.1093/mp/sst009, PMID: 23335733

[ref15] Egea I. Pineda B. Ortiz-Atienza A. Plasencia F. A. Drevensek S. Garcia-Sogo B.. (2018). The SlCBL10 calcineurin B-like protein ensures plant growth under salt stress by regulating Na^+^ and Ca^2+^ homeostasis. Plant Physiol. 176, 1676–1693. doi: 10.1104/pp.17.01605, PMID: 29229696 PMC5813568

[ref16] Gong Z. Z. Xiong L. M. Shi H. Z. Yang S. H. Herrera-Estrella L. R. Xu G. H.. (2020). Plant abiotic stress response and nutrient use efficiency. Sci. China Life Sci. 63, 635–674. doi: 10.1007/s11427-020-1683-x, PMID: 32246404

[ref17] Gutiérrez-Beltrán E. Personat J. M. de la Torre F. Del Pozo O. (2017). A universal stress protein involved in oxidative stress is a phosphorylation target for protein kinase CIPK6. Plant Physiol. 173, 836–852. doi: 10.1104/pp.16.00949, PMID: 27899535 PMC5210712

[ref18] Hajiboland R. (2014). “Reactive oxygen species and photosynthesis,” in Oxidative Damage to Plants. ed. Ahmad P. (USA: Elsevier), 1–63.

[ref19] Horsch R. B. Fry J. E. Hoffmann N. L. Eichholtz D. Rogers S. G. Fraley R. T. (1985). A simple and general method for transferring genes into plants. Science 227, 1229–1231. 17757866 10.1126/science.227.4691.1229

[ref20] Hu D. G. Li M. Luo H. Dong Q. L. Yao Y. X. You C. X.. (2012). Molecular cloning and functional characterization of *MdSOS2* reveals its involvement in salt tolerance in apple callus and *Arabidopsis*. Plant Cell Rep. 31, 713–722. doi: 10.1007/s00299-011-1189-5, PMID: 22108717

[ref21] Ismail A. M. Horie T. (2017). Genomics, physiology, and molecular breeding approaches for improving salt tolerance. Annu. Rev. Plant Biol. 68, 405–434. doi: 10.1146/annurev-arplant-042916-040936, PMID: 28226230

[ref502] Kanehisa M. Furumichi M. Sato Y. Ishiguro-Watanabe M. Tanabe M. (2020). KEGG: integrating viruses and cellular organisms. Nucleic Acids Res. 49, D545–D551. PMID: 33125081 10.1093/nar/gkaa970 PMC7779016

[ref22] Kim B.-G. Waadt R. Cheong Y. H. Pandey G. K. Dominguez-Solis J. R. Schültke S.. (2007). The calcium sensor CBL10 mediates salt tolerance by regulating ion homeostasis in *Arabidopsis*. Plant J. 52, 473–484. doi: 10.1111/j.1365-313X.2007.03249.x, PMID: 17825054

[ref23] Kissoudis C. (2016). Genetics and regulation of combined abiotic and biotic stress tolerance in tomato. PhD thesis. Wageningen University.

[ref24] Knight H. Knight M. R. (2001). Abiotic stress signalling pathways: specificity and cross-talk. Trends Plant Sci. 6, 262–267. doi: 10.1016/S1360-1385(01)01946-X, PMID: 11378468

[ref25] Kurusu T. Hamada J. Nokajima H. Kitagawa Y. Kiyoduka M. Takahashi A.. (2010). Regulation of microbe-associated molecular pattern-induced hypersensitive cell death, phytoalexin production, and defense gene expression by calcineurin B-like protein-interacting protein kinases, OsCIPK14/15, in rice cultured cells. Plant Physiol. 153, 678–692. doi: 10.1104/pp.109.151852, PMID: 20357140 PMC2879771

[ref26] Lamers J. van der Meer T. Testerink C. (2020). How plants sense and respond to stressful environments. Plant Physiol. 182, 1624–1635. doi: 10.1104/pp.19.01464, PMID: 32132112 PMC7140927

[ref27] Lapin D. Bhandari D. D. Parker J. E. (2020). Origins and immunity networking functions of EDS1 family proteins. Annu. Rev. Phytopathol. 58, 253–276. doi: 10.1146/annurev-phyto-010820-012840, PMID: 32396762

[ref28] Letunic I. Bork P. (2018). 20 years of the SMART protein domain annotation resource. Nucleic Acids Res. 46, D493–D496. doi: 10.1093/nar/gkx922, PMID: 29040681 PMC5753352

[ref29] Li L. Kim B.-G. Cheong Y. H. Pandey G. K. Luan S. (2006). A Ca^2+^ signaling pathway regulates a K^+^ channel for low-K response in *Arabidopsis*. Proc. Nati. Acad. Sci. U. S. A. 103, 12625–12630. doi: 10.1073/pnas.0605129103, PMID: 16895985 PMC1567929

[ref30] Li D. D. Xia X. L. Yin W. L. Zhang H. C. (2012a). Two poplar calcineurin B-like proteins confer enhanced tolerance to abiotic stresses in transgenic *Arabidopsis thaliana*. Biol. Plant. 57, 70–78. doi: 10.1007/s10535-012-0251-7

[ref31] Li Z. Y. Xu Z. S. He G. Y. Yang G. X. Chen M. Li L. C.. (2012b). Overexpression of soybean *GmCBL1* enhances abiotic stress tolerance and promotes hypocotyl elongation in *Arabidopsis*. Biochem. Biophys. Res. Commun. 427, 731–736. doi: 10.1016/j.bbrc.2012.09.128, PMID: 23044418

[ref32] Lin H. X. Yang Y. Q. Quan R. D. Mendoza I. Wu Y. S. Du W. M.. (2009). Phosphorylation of SOS3-LIKE CALCIUM BINDING PROTEIN8 by SOS2 protein kinase stabilizes their protein complex and regulates salt tolerance in *Arabidopsis*. Plant Cell 21, 1607–1619. doi: 10.1105/tpc.109.066217, PMID: 19448033 PMC2700523

[ref503] Ling Q. H. Huang W. H. Jarvis P. (2011). Use of a SPAD-502 meter to measure leaf chlorophyll concentration in Arabidopsis thaliana. Photosyn Res. 107, 209–214. PMID: 21188527 10.1007/s11120-010-9606-0

[ref33] Liu P. Duan Y. H. Liu C. Xue Q. H. Guo J. Qi T.. (2018). The calcium sensor TaCBL4 and its interacting protein TaCIPK5 are required for wheat resistance to stripe rust fungus. J. Exp. Bot. 69, 4443–4457. doi: 10.1093/jxb/ery227, PMID: 29931351

[ref34] Liu P. Guo J. Zhang R. M. Zhao J. X. Liu C. Qi T.. (2019). TaCIPK10 interacts with and phosphorylates TaNH2 to activate wheat defense responses to stripe rust. Plant Biotechnol. J. 17, 956–968. doi: 10.1111/pbi.13031, PMID: 30451367 PMC6587807

[ref501] Livak K. J. Schmittgen T. D. (2001). Analysis of relative gene expression data using real-time quantitative PCR and the 2-ΔΔCT method. Methods. 25, 402–408. 11846609 10.1006/meth.2001.1262

[ref35] Lorenzo O. Piqueras R. Sanchez-Serrano J. J. Solano R. (2003). ETHYLENE RESPONSE FACTOR1 integrates signals from ethylene and jasmonate pathways in plant defense. Plant Cell 15, 165–178. doi: 10.1105/tpc.007468, PMID: 12509529 PMC143489

[ref36] Luan S. (2009). The CBL-CIPK network in plant calcium signaling. Trends Plant Sci. 14, 37–42. doi: 10.1016/j.tplants.2008.10.005, PMID: 19054707

[ref37] Ludwig A. A. Tenhaken R. (2001). A new cell wall located N-rich protein is strongly induced during the hypersensitive response in *Glycine max* L. Eur. J. Plant Pathol. 107, 323–336. doi: 10.1023/A:1011202225323

[ref38] Lv F. L. Zhang H. C. Xia X. L. Yin W. L. (2014). Expression profiling and functional characterization of a CBL-interacting protein kinase gene from *Populus euphratica*. Plant Cell Rep. 33, 807–818. doi: 10.1007/s00299-013-1557-4, PMID: 24413762

[ref39] Ma X. Gai W. X. Qiao Y. M. Ali M. Wei A. M. Luo D. X.. (2019). Identification of CBL and CIPK gene families and functional characterization of CaCIPK1 under *Phytophthora capsici* in pepper (*Capsicum annuum* L.). BMC Genomics 20:775. doi: 10.1186/s12864-019-6125-z, PMID: 31653202 PMC6814991

[ref40] Ma X. Li Q. H. Yu Y. N. Qiao Y. M. Haq S. U. Gong Z. H. (2020). The CBL-CIPK pathway in plant response to stress signals. Int. J. Mol. Sci. 21:5668. doi: 10.3390/ijms21165668, PMID: 32784662 PMC7461506

[ref41] Manishankar P. Wang N. Koster P. Alatar A. A. Kudla J. (2018). Calcium signaling during salt stress and in the regulation of ion homeostasis. J. Exp. Bot. 69, 4215–4226. doi: 10.1093/jxb/ery201, PMID: 29800460

[ref42] Martinez-Atienza J. Jiang X. Garciadeblas B. Mendoza I. Zhu J. K. Pardo J. M.. (2007). Conservation of the salt overly sensitive pathway in rice. Plant Physiol. 143, 1001–1012. doi: 10.1104/pp.106.092635, PMID: 17142477 PMC1803719

[ref43] Martinoia E. Maeshima M. Neuhaus H. E. (2007). Vacuolar transporters and their essential role in plant metabolism. J. Exp. Bot. 58, 83–102. doi: 10.1093/jxb/erl183, PMID: 17110589

[ref44] Miller G. Suzuki N. Ciftci-Yilmaz S. Mittler R. (2010). Reactive oxygen species homeostasis and signalling during drought and salinity stresses. Plant Cell Environ. 33, 453–467. doi: 10.1111/j.1365-3040.2009.02041.x, PMID: 19712065

[ref45] Munns R. (2005). Genes and salt tolerance: bringing them together. New Phytol. 167, 645–663. doi: 10.1111/j.1469-8137.2005.01487.x, PMID: 16101905

[ref46] Munns R. Tester M. (2008). Mechanisms of salinity tolerance. Annu. Rev. Plant Biol. 59, 651–681. doi: 10.1146/annurev.arplant.59.032607.092911, PMID: 18444910

[ref47] Nejat N. Mantri N. (2017). Plant immune system: grosstalk between responses to biotic and abiotic stresses the missing link in understanding plant defence. Curr. Issues Mol. Biol. 23, 1–16. doi: 10.21775/cimb.023.001, PMID: 28154243

[ref48] Perochon A. Aldon D. Galaud J. P. Ranty B. (2011). Calmodulin and calmodulin-like proteins in plant calcium signaling. Biochimie 93, 2048–2053. doi: 10.1016/j.biochi.2011.07.012, PMID: 21798306

[ref49] Pertea M. Pertea G. M. Antonescu C. M. Chang T. C. Mendell J. T. Salzberg S. L. (2015). StringTie enables improved reconstruction of a transcriptome from RNA-seq reads. Nat. Biotechnol. 33, 290–295. doi: 10.1038/nbt.3122, PMID: 25690850 PMC4643835

[ref50] Pontler D. Godiard L. Marco Y. Roby D. (1994). *hsr203J*, a tobacco gene whose activation is rapid, highly localized and specific for incompatible plant/pathogen interactions. Plant J. 5, 507–521. doi: 10.1046/j.1365-313X.1994.5040507.x 8012404

[ref51] Prelich G. (2012). Gene overexpression: uses, mechanisms, and interpretation. Genetics 190, 841–854. doi: 10.1534/genetics.111.136911, PMID: 22419077 PMC3296252

[ref52] Qiu Q. S. Guo Y. Dietrich M. A. Schumaker K. S. Zhu J. K. (2002). Regulation of SOS1, a plasma membrane Na^+^/H^+^ exchanger in *Arabidopsis thaliana*, by SOS2 and SOS3. Proc. Nati. Acad. Sci. U. S. A. 99, 8436–8441. doi: 10.1073/pnas.122224699 PMC123085 12034882

[ref53] Qiu Q. S. Guo Y. Quintero F. J. Pardo J. M. Schumaker K. S. Zhu J. K. (2004). Regulation of vacuolar Na^+^/H^+^ exchange in *Arabidopsis thaliana* by the salt-overly-sensitive (SOS) pathway. J. Biol. Chem. 279, 207–215. doi: 10.1074/jbc.M307982200, PMID: 14570921

[ref54] Quan R. Lin H. Mendoza I. Zhang Y. Cao W. Yang Y.. (2007). SCABP8/CBL10, a putative calcium sensor, interacts with the protein kinase SOS2 to protect Arabidopsis shoots from salt stress. Plant Cell 19, 1415–1431. doi: 10.1105/tpc.106.042291, PMID: 17449811 PMC1913747

[ref55] Ragel P. Ródenas R. García-Martín E. Andrés Z. Villalta I. Nieves-Cordones M.. (2015). CIPK23 regulates HAK5-mediated high-affinity K^+^ uptake in *Arabidopsis* roots. Plant Physiol. 169, 2863–2873. doi: 10.1104/pp.15.01401, PMID: 26474642 PMC4677917

[ref56] Ray S. K. Macoy D. M. Kim W. Y. Lee S. Y. Kim M. G. (2019). Role of RIN4 in regulating PAMP-triggered immunity and effector-triggered immunity: current status and future perspectives. Mol. Cell 42, 503–511. doi: 10.14348/molcells.2019.2433, PMID: 31362467 PMC6681865

[ref57] Saijo Y. Loo E. P. (2020). Plant immunity in signal integration between biotic and abiotic stress responses. New Phytol. 225, 87–104. doi: 10.1111/nph.15989, PMID: 31209880

[ref58] Sánchez-Barrena M. J. Martínez-Ripoll M. Albert A. (2013). Structural biology of a major signaling network that regulates plant abiotic stress: the CBL-CIPK mediated pathway. Int. J. Mol. Sci. 14, 5734–5749. doi: 10.3390/ijms14035734, PMID: 23481636 PMC3634423

[ref59] Sardar A. Nandi A. K. Chattopadhyay D. (2017). CBL-interacting protein kinase 6 negatively regulates immune response to *Pseudomonas syringae* in *Arabidopsis*. J. Exp. Bot. 68, 3573–3584. doi: 10.1093/jxb/erx170, PMID: 28541442 PMC5853215

[ref60] Satir O. Berberoglu S. (2016). Crop yield prediction under soil salinity using satellite derived vegetation indices. Field Crop Res. 192, 134–143. doi: 10.1016/j.fcr.2016.04.028

[ref504] Schmidt G. W. Delaney S. K. (2010). Stable internal reference genes for normalization of real-time RT-PCR in tobacco (Nicotiana tabacum) during development and abiotic stress. Mol. Genet. Genomics. 283, 233–241. 20098998 10.1007/s00438-010-0511-1

[ref61] Shi S. J. An L. L. Mao J. J. Aluko O. O. Ullah Z. Xu F. Z.. (2021). The CBL-interacting protein kinase NtCIPK23 positively regulates seed germination and early seedling development in tobacco (*Nicotiana tabacum* L.). Plants 10:323. doi: 10.3390/plants10020323, PMID: 33567573 PMC7915007

[ref62] Shi H. Z. Quintero F. J. Pardo J. M. Zhu J. K. (2002). The putative plasma membrane Na^+^/H^+^ antiporter SOS1 controls long-distance Na^+^ transport in plants. Plant Cell 14, 465–477. doi: 10.1105/tpc.010371, PMID: 11884687 PMC152925

[ref63] Sinha M. Singh R. P. Kushwaha G. S. Iqbal N. Singh A. Kaushik S.. (2014). Current overview of allergens of plant pathogenesis related protein families. Sci. World J. 2014:543195. doi: 10.1155/2014/543195, PMID: 24696647 PMC3947804

[ref64] Snedden W. A. Fromm H. (1998). Calmodulin, calmodulin-related proteins and plant responses to the environment. Trends Plant Sci. 3, 299–304. doi: 10.1016/S1360-1385(98)01284-9

[ref65] Snedden W. A. Fromm H. (2001). Calmodulin as a versatile calcium signal transducer in plants. New Phytol. 151, 35–66. doi: 10.1046/j.1469-8137.2001.00154.x, PMID: 33873389

[ref66] Steinhorst L. Mähs A. Ischebeck T. Zhang C. Zhang X. Arendt S.. (2015). Vacuolar CBL-CIPK12 Ca^2+^-sensor-kinase complexes are required for polarized pollen tube growth. Curr. Biol. 25, 1475–1482. doi: 10.1016/j.cub.2015.03.053, PMID: 25936548

[ref67] Sun H. Sun X. Wang H. Ma X. (2020). Advances in salt tolerance molecular mechanism in tobacco plants. Hereditas 157:5. doi: 10.1186/s41065-020-00118-0, PMID: 32093781 PMC7041081

[ref68] Tang Y. Gao C. C. Gao Y. Yang Y. Shi B. Yu J. L.. (2020). OsNSUN2-mediated 5-methylcytosine mRNA modification enhances rice adaptation to high temperature. Dev. Cell 53, 272–286. doi: 10.1016/j.devcel.2020.03.009, PMID: 32275888

[ref69] Tang R. J. Liu H. Bao Y. Lv Q. D. Yang L. Zhang H. X. (2010). The woody plant poplar has a functionally conserved salt overly sensitive pathway in response to salinity stress. Plant Mol. Biol. 74, 367–380. doi: 10.1007/s11103-010-9680-x, PMID: 20803312

[ref70] Tang R. J. Yang Y. Yang L. Liu H. Wang C. T. Yu M. M.. (2014). Poplar calcineurin B-like proteins PtCBL10A and PtCBL10B regulate shoot salt tolerance through interaction with PtSOS2 in the vacuolar membrane. Plant Cell Environ. 37, 573–588. doi: 10.1111/pce.12178, PMID: 23941462

[ref71] Tang R.-J. Zhao F.-G. Garcia V. J. Kleist T. J. Yang L. Zhang H.-X.. (2015). Tonoplast CBL-CIPK calcium signaling network regulates magnesium homeostasis in *Arabidopsis*. Proc. Nati. Acad. Sci. U. S. A. 112, 3134–3139. doi: 10.1073/pnas.1420944112, PMID: 25646412 PMC4364200

[ref72] Tran B. Q. Jung S. (2020). Modulation of chloroplast components and defense responses during programmed cell death in tobacco infected with *Pseudomonas syringae*. Biochem. Biophys. Res. Commun. 528, 753–759. doi: 10.1016/j.bbrc.2020.05.086, PMID: 32527587

[ref73] Trewavas A. J. Malhό R. (1998). Ca^2+^ signalling in plant cells: the big network! Curr. Opin. Plant Biol. 1, 428–433. doi: 10.1016/S1369-5266(98)80268-9, PMID: 10066614

[ref74] Van Zelm E. Zhang Y. X. Testerink C. (2020). Salt tolerance mechanisms of plants. Annu. Rev. Plant Biol. 71, 403–433. doi: 10.1146/annurev-arplant-050718-100005, PMID: 32167791

[ref75] Wang J. Ye B. Q. Yin J. J. Yuan C. Zhou X. G. Li W. T.. (2015). Characterization and fine mapping of a light-dependent *leaf lesion mimic mutant 1* in rice. Plant Physiol. Biochem. 97, 44–51. doi: 10.1016/j.plaphy.2015.09.001, PMID: 26410574

[ref76] Wang C. T. Yin X. L. Kong X. X. Li W. S. Ma L. Sun X. D.. (2013). A series of TA-based and zero-background vectors for plant functional genomics. PLoS One 8:e59576. doi: 10.1371/journal.pone.0085650, PMID: 23555713 PMC3612078

[ref77] Waterhouse A. Bertoni M. Bienert S. Studer G. Tauriello G. Gumienny R.. (2018). SWISS-MODEL: homology modelling of protein structures and complexes. Nucleic Acids Res. 46, W296–W303. doi: 10.1093/nar/gky427, PMID: 29788355 PMC6030848

[ref78] Yan Y. He X. Y. Hu W. Liu G. Y. Wang P. He C. Z.. (2018). Functional analysis of MeCIPK23 and MeCBL1/9 in cassava defense response against *Xanthomonas axonopodis* pv. *Manihotis*. Plant Cell Rep. 37, 887–900. doi: 10.1007/s00299-018-2276-7, PMID: 29523964

[ref79] Yin X. C. Xia Y. Q. Xie Q. Cao Y. X. Wang Z. Y. Hao G. P.. (2019). CBL10-CIPK8-SOS1, A novel SOS pathway, functions in Arabidopsis to regulate salt tolerance. J. Exp. Bot. 71, 1–14. doi: 10.1093/jxb/erz549 PMC7242078 31858132

[ref80] Yukawa M. Tsudzuki T. Sugiura M. (2006). The chloroplast genome of *Nicotiana sylvestris* and *Nicotiana tomentosiformis*: complete sequencing confirms that the *Nicotiana sylvestris* progenitor is the maternal genome donor of *Nicotiana tabacum*. Mol. Gen. Genomics. 275, 367–373. doi: 10.1007/s00438-005-0092-6, PMID: 16435119

[ref81] Zaman M. Heng S. A. S. L. (2018). Guideline for Salinity Assessment, Mitigation and Adaptation Using Nuclear and Related Techniques. Switzerland: Springer Nature.

[ref82] Zhang H. C. Lv F. L. Han X. Xia X. L. Yin W. L. (2013). The calcium sensor PeCBL1, interacting with PeCIPK24/25 and PeCIPK26, regulates Na^+^/K^+^ homeostasis in *Populus euphratica*. Plant Cell Rep. 32, 611–621. doi: 10.1007/s00299-013-1394-5, PMID: 23423605

[ref83] Zhao J. Barkla B. J. Marshall J. Pittman J. K. Hirschi K. D. (2007). The *Arabidopsis cax3* mutants display altered salt tolerance, pH sensitivity and reduced plasma membrane H^+^-ATPase activity. Planta 227, 659–669. doi: 10.1007/s00425-007-0648-2, PMID: 17968588

[ref84] Zulfugarov I. S. Tovuu A. Eu Y.-J. Dogsom B. Poudyal R. S. Nath K.. (2014). Production of superoxide from photosystem II in a rice (*Oryza sativa* L.) mutant lacking PsbS. BMC Plant Biol. 14:242. doi: 10.1186/s12870-014-0242-2, PMID: 25342550 PMC4219129

