# Supplementary material for: Overexpression of NtCBL5A Leads to Necrotic Lesions by Enhancing Na+ Sensitivity of Tobacco Leaves Under Salt Stress
Source: Front Plant Sci. 2021 Sep 17;12:740976. doi: 10.3389/fpls.2021.740976 (PMC8484801; doi:10.3389/fpls.2021.740976)
Supplement: Supplementary file 1 [file Data_Sheet_1.PDF]

TABLE S1 | Primers used for gene expression analysis and plasmid construction

Primer name	Sequence (5' to 3')	Description
NtCBL5A-1F	ATGGGCTGTGCTTTAAGAAAGCAAGA	Used for the clone of *NtCBL5A* CDS
NtCBL5A-1R	TCAGAAATCCTTGTAAATCTCATC	
NtCBL5A-3F-*Sac* Ⅰ	CGAGCTCATGGGCTGTGCTTTAAGAAAGCAAGA	Used for the construction of plasmid pCHF3-NtCBL5A
NtCBL5A-3R-*Kpn* Ⅰ	GGGGTACCTCAGAAATCCTTGTAAATCTCATC	
NtCBL5Apro-1F-*Sal* Ⅰ	ACGCGTCGACGTTAGATTTGTCCCTCCGTG	Used for the construction of plasmid pBI101-NtCBL5Apro::GUS
NtCBL5Apro-1R-*Sma* Ⅰ	TCCCCCGGGTTTATAGTAATATTTTTGTGTTGTTATG	
pCHF3-R	ATTCTGGTGTGTGCGCAATG	For the indentification of *NtCBL5A*-OE transgenic tobacco.
pBI101-F	CCGATTCATTAATGCAGCTG	For the indentification of *NtCBL5Apro::GUS* transgenic tobacco.
NtCBL5A-UTR-31F	TTTGCTAATAAACCCATCTTTG	For the identification of endogenous *NtCBL5A*, NtCBL5A-UTR-31F was designed based on the 5'-UTR sequence of *NtCBL5A*
NtCBL5A-81R	TCCAAAGAAAAATGTGTCTGAG	
NtCBL5A-564F	CTCAAGGATATCACAGCTGCATT	For the identification of exogenous *NtCBL5A*, pCHF3-Allcheck-1 was designed based on the sequence of 3'-UTR of exogenous *NtCBL5A*, referring to pCHF3-Allcheck-2 (Shi et al., 2021).
pCHF3-Allcheck-1	TGCAGGTCGACTCTAGAGGAT	
NRP-qF	ACTATGTTTCCAAAAGGCCTGA	RT-qPCR primers for *NRP*
NRP-qR	CCATTTAAGCCAAAATCAAGAACC	(Nitab4.5_0000798g0120)
HSR203J-qF	CTTGCGATGCCGGTTTTTCC	RT-qPCR primers for *HSR203J*
HSR203J-qR	GGCGAGTCGCATTGGAGATA	(Nitab4.5_0002719g0120)
PR1a-qF	GTAATATCCCACTCTTGCCGTG	RT-qPCR primers for *PR1a*
PR1a-qR	CCTCAGCTAGGTTTTCGCCG	(Nitab4.5_0003771g0010)
PR1b-qF	ACCATTAACTTGGGACAACGG	RT-qPCR primers for *PR1b*
PR1b-qR	TACGCCAAACCACCTGAGTA	(Nitab4.5_0005400g0020)
PR1c-qF	ATCCCACTCTTGTCATGCTC	RT-qPCR primers for *PR1c*
PR1c-qR	GAAATCGCCACTTCCCCAAG	(Nitab4.5_0004861g0040)
PR-Q-qF	GTGGATCCCTGAGTGCAGAA	RT-qPCR primers for *PR-Q*
PR-Q-qR	CGTGGGAAGATGGCTTGTTG	(Nitab4.5_0003207g0080)
PR-R minor-qF	AACTAATGGCGGTTGCCGTA	RT-qPCR primers for *PR-R minor*
PR-R minor-qR	CCACATGATCCAGGCCCATT	(Nitab4.5_0004097g0050)
PR-R major-qF	GTGAACCCAGGAACAGTCCA	RT-qPCR primers for *PR-R major*
PR-R major-qR	ACGACATCCTCCATTGGTCG	(Nitab4.5_0000360g0100)
EDS1-qF	AGGCCGAAGCGTTATAGGTT	RT-qPCR primers for *EDS1*
EDS1-qR	GGGACCAATCCCATGCCTTT	(Nitab4.5_0002101g0050)
RIN4-qF	GGGATGAACCAATGGGGCTA	RT-qPCR primers for *RIN4*
RIN4-qR	TTCCCTTCCAAAACAGGCGA	(Nitab4.5_0003020g0010)
ERF1-qF	GCTTCCTCAAGTACTCCACACA	RT-qPCR primers for *ERF1*
ERF1-qR	TGTAACGGTGATTGATCTTGGA	(Nitab4.5_0010541g0010), ERF1-qF was designed from 5'-UTR
CAT1-qF	TGTTCAAGTACTGTGGTCATCTTCT	RT-qPCR primers for *CAT1*

CAT1-qR	AACGGAAGACAGAGTAGCAGC	(Nitab4.5_0009821g0010), CAT1-qF and CAT1-qR were designed from 3’-UTR
CHX18-qF	CAGCACCCCTGAAATGTCCA	RT-qPCR primers for *NHX18*
CHX18-qR	ACAACACGTGGCTGTCTCAA	(Nitab4.5_0006998g0030)
NCX1-qF	CCGAGAAGAGAAGTGCTTGGA	RT-qPCR primers for *NCX1*
NCX1-qR	TCTTGGCAAGTCAAGTGGCA	(Nitab4.5_0005404g0030)
CAX3-qF	TGTCTGTGGAACCAGTACTTAACA	RT-qPCR primers for *CAX3*
CAX3-qR	ATAACCGCCTCATCTTCCGC	(Nitab4.5_0000102g0080)
CNGC1-qF	TCCTGATAATCTGAGAGCACGA	RT-qPCR primers for *CNGC1*
CNGC1-qR	AGTCGACCACACATTGCATCT	(Nitab4.5_0000258g0120)
PsaH-qF	CCATTGCCGGAACAAAACTCA	RT-qPCR primers for *PsaH*
PsaH-qR	GAACTTGCTCTGAAGGGGGT	(Nitab4.5_0000351g0060)
PsaE-qF	AGCAGAGACAAAGACATGGCAA	RT-qPCR primers for *PsaE*
PsaE-qR	AACGAGCCTCGGGAAGTTG	(Nitab4.5_0000385g0230), PsaE-qF was designed from 5’-UTR
PsaD-qF	TGGCAACTCAAGCTTCTCTCTT	RT-qPCR primers for *PsaD*
PsaD-qR	CTTCTTTTGTGGCGGCTTCTT	(Nitab4.5_0014875g0010)
PsbQ-qF	TCCCCCACCCTACTTCTATCA	RT-qPCR primers for *PsbQ*
PsbQ-qR	TAACAGTGCTCAAACGGGCT	(Nitab4.5_0002345g0070), PsbQ-qF was designed from 5’-UTR
PsbX-qF	TGCCAATAAGGCCATCAAAACAAA	RT-qPCR primers for *PsbX*
PsbX-qR	CAGCAGCTTGTGCTACATCAG	(Nitab4.5_0000073g0060)
OEE1-qF	GGAGCTCCAGAAGGAGAACG	RT-qPCR primers for *OEE1*
OEE1-qR	ACCCCAAGGTCTTAAAAATCTCTCT	(Nitab4.5_0000108g0110), OEE1-qR was designed from 3’-UTR
Lhca3-qF	TGATCATTTGGCTGATCCCGT	RT-qPCR primers for *Lhca3*
Lhca3-qR	CCCCTTTTTAGCATTACAGATGACA	(Nitab4.5_0000923g0200), Lhca3-qR was designed from 3’-UTR
Lhcb3-qF	TGTCACTGGCAAAGGTCCTC	RT-qPCR primers for *Lhcb3*
Lhcb3-qR	GCTAGTGGTATTTATCACATGGCA	(Nitab4.5_0012832g0010), Lhcb3-qR was designed from 3’-UTR
Lhcb4-qF	CCCAACGCACCTTGTTTTTCA	RT-qPCR primers for *Lhcb4*
Lhcb4-qR	TAGCGTTTGATATTTCCTTCTCCT	(Nitab4.5_0011597g0020), both Lhcb4-qF and Lhcb4-qR were designed from 3’-UTR
Fd-qF	TCCTTACTCATGCAGAGCTGG	RT-qPCR primers for *Fd*
Fd-qR	ATTGAGGGGTTTAGGCAGTGA	(Nitab4.5_0004129g0010)
F-ATPase delta subunit-qF	CAGTCCAGATCACCCCCGA	RT-qPCR primers for *F-ATPase delta subunit*
F-ATPase delta subunit -qR	TAGGGTTCCGTTCGACTTGG	(Nitab4.5_0006745g0030)
GAPA-qF	ACACTGGTGGTGTCAAGCAA	RT-qPCR primers for *GAPDH*
GAPA-qR	TCTTGGCTCCAGCCTGAATG	(Nitab4.5_0010299g0040)
