# Supplementary material for: Overexpression of NtCBL5A Leads to Necrotic Lesions by Enhancing Na+ Sensitivity of Tobacco Leaves Under Salt Stress
Source: Front Plant Sci. 2021 Sep 17;12:740976. doi: 10.3389/fpls.2021.740976 (PMC8484801; doi:10.3389/fpls.2021.740976)
Supplement: Supplementary file 2 [file Data_Sheet_2.PDF]

A

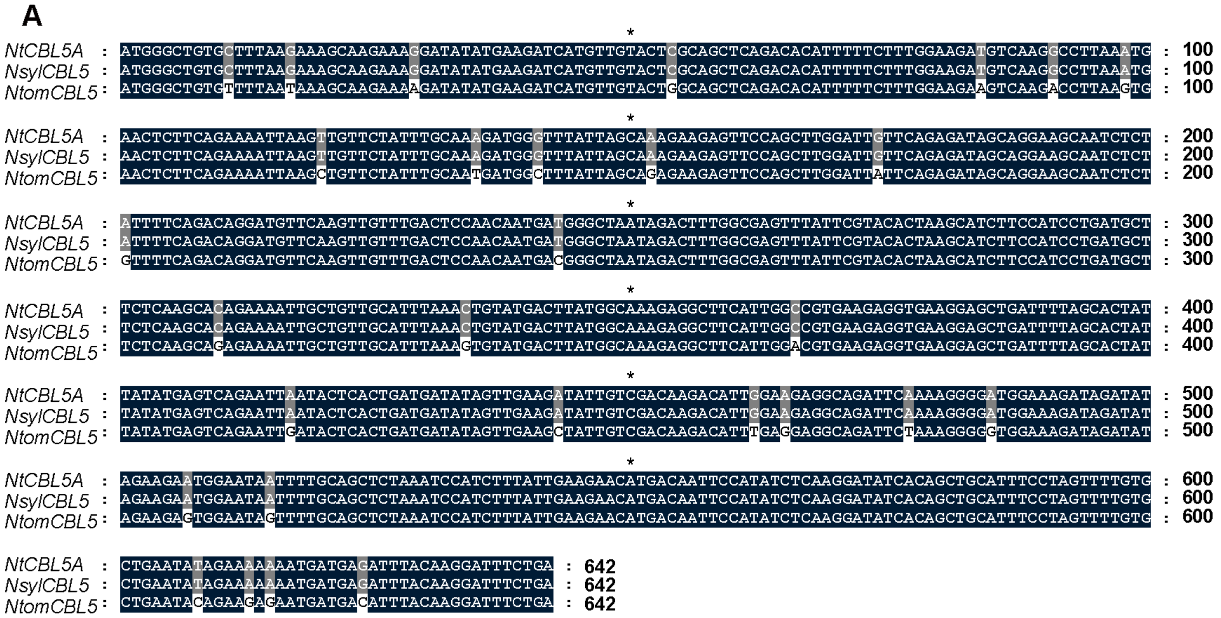

B

```
-2780                       GTTAGATTTG TCCCTCCGTG TTAATTTATG TAGCATAGTT TGATTAGGCA CGAAATTTAA GAACTAAATA AAAAAAAATT
-2700 GAAATTTGTC ATCTTGAATG TGTCATAAGA TTTCCTAAAG GCAGTCTGGT GCACTAAGCT CTCGCTATGT GCGGAGTCCG GGGAAGGGTC GCGCCACAAA
-2600 GATCTATTGT ACGCAGTCTT ACCTTACATT TCTACAAGAG GTTGTTTCCA CGGCTCGAAC CCGTGACCTC CTGATCGCAT GGCAGCAACT TTACCAGTTA
-2500 CGCCAAGGCT CCCCTTCTGC TAGAGGCGGA TCCAGGATTT ATATCCTATG GGTTTAGTCT TTAAGATATT TAGCATTGAA CTCATTATAC TTTTAAAGCT
-2400 ATGAGTTCAA ATTACTATTT GTTGCAATTT TAGTAAAATT TATACATAAA TTTATGTTCC GCATTAAAAA TTATGGGTTC AGTTGAATCC ATCACATATA
-2300 CGCTACATAC GCCGCTACCT TCTGCCACGA GAGTTGCTAT GGTTTATAAA ATTATGCCAA TGATGGAGGA CGGGTCAAGC AGAACTGGCA AGATTCTAAG
-2200 CTGACAAGTT TCTAAAGAAG GTGCGCACAT ATTATTTTAG GCGAATCTAG TGGTTATGAT CTAGATATTC TTGCTTAATA TAGGTAGAAG GCGAAAAATA
-2100 AGTTAGAAAA ATTAATTGGA GGAATATATT CTAAGTAGGA TTTGAAATTT TATGCAAAGA TTATATGAAT GATAACGAGA GAGATCTCTA TTTAGATGGA
-2000 TGATAGGAAA GACAATAACT TAATGCTAGT ACACTCCTTA CCTCGTAGTA TTAAATGAAT GATTATGTCA AAATAGATGT TTGGTGGAGG GGCGGATTTA
-1900 AAATTTAAAT TTGATGGATT AATTTTTTAG TTCTACTTAT CATTATATTT ATTATACTTT CTAAAATTAG GGGTTAAAAT AATTAATATT TTAAAATTCT
-1800 AGAGATTTTT CACATATATA TTTATACTCC GATTGAAAAT ATATATTGAA TTCACTTGAA CCCGCTAATT ATATGCTACA TCCACCATTA GCCTAATTTA
-1700 TGGTTGTCAT TATCCTTTTG GAATTTATTG CTTATCTCAT TTTAGCTTTT CAAAAATTCA AGGTGCTTAA ATCTCTATTT TCCCTGCATC TTTTAATTTT
-1600 CTAATCTTTT ATGTAAGAAT TTAAATGTTT CGATTGTTGG GGGTACGAAG AAAGTCCTAC ATCAGTGGCT GAAATTATTG AAAGAAAAAA GTCCTACATT
-1500 AATGACTGAA ATTAAATTCA ACAAATATAA GGAAGCAAGT CGTAGGCCTC ACTATCTATC AATCAAGAAG GTAAAGTTTG GAAAAAATCG TTGGGTTTGG
-1400 TTTAAGCGGA CAATATTATA TTATGTAAGA GTACAGTATC CTTGGGCTTG TTTAGCCCAA CAAATGGTAT CAAAGCTCAT ATTCGGCGGA ACAAGTATGG
-1300 CAATAATAGA GTGATGGTAC GGGGCTTAGT TTGCGTATTT TCATTTAGTA ATGATATTTG TTGAGCGATA TGACCAATGA CAATTAATAC AACCGACTTC
-1200 AACTTATTTG AAATTGAAGC GTAATTATTG CTATTGTAGC ACTGACCTTT GAGCTTTAAT ACTTTACCGA TTCTTGAGAA AATAATTCAT GAATTTGGAA
-1100 CATTGGGAAT TACTTATGTA CAACCAATTT GGTGAATGTG AACAATTCTG TTCCTTACAA CCGCACATTA TAAAAGCAAC AGCACCTGGT TCCACATTAG
-1000 CTGAACAACA TTAAAGAATA AAGGAAATTA CCTGAATGAA AATGATAACC TAAAGAATAT AATGTACCAT TTGAAATCTC TAAAAATTTT GTGTCGAGGG
-900  TCTTTCGGAA ACAGCTTATT TCCGAGGCAG GGGTAAGGTT TGCGTATACA CTACTCTCCG CAGACCCCAC ACGTGGGATT ATACTGGGTT GTTGTTGTTG
-800  TTATTGTTGA AATCTCTAAA ATTTTTGTGT CGAGGGTCTT TCGGAAACAG CCTATTTTCG GGGCAGGGGT AAGGTTTGCA TAAACACTAC TCTCCGCAGA
-700  CCCGACATGT GGGATTATAC TGGGTTGTTG TTGTTGTTAT TGTTGAAATC TCTAAATACC TTTAATAGAA TGTACACTTT GCTCTTAATT ATAGGGCCAG
-600  TAGCTGAATT TCTCTAAGGA CGTTCAAGAT TTAGTATATA TGTAGAAGAA GTAACATTAA CCTATATATC CAGTATAATT TTACGTTAGC TATTGCTTCG
-500  CCCATGTATA TTTTTTGTAC ATTTTACTAA GTCACTCCCA CAATTTTTTC CACTAAAAAA TAATTACACC TTATATTAGT CTCAAACTAG TTGGGGTCAT
-400  ACCTCGCTTA TTAATTTGAC CATTTTTGAC GTACTCAAAT TTAACTCAAC CTGCTCATTT GTTACCTTTC TATGGTCAAG AAAGTAAGGT AAAGGGCTTA
-300  TGAACGTTTG GCTATGCTTT TTATCCATGC AATCAAATCC GCTGCACAAG GTATTCTGCG TTCATGCAGT GTCCGAGAAA GGACCGCAAC TCAAGGGGTA
-200  TGATGTAGAC AGCCTACCCT AATGCAAGCA TTAGTGACTG CTTTTACGGC TTGAACATGT GACCTATAGA ATACACGGAG CTAACTTCAT CGTTGCTCTA
-100  AAGCTCCCTT TCCATCTATA CAATCAAACC CCTCTTTATA TATAGCCTAC ACAAAACAGA TAAATGTGAG AGCATAACAA CACAAAAATA TTACTATAAA
+1    ATG
```

Start condon

FIGURE S1 | The coding sequence alignment of *NtCBL5A*, *N. sylvestris CBL5* (*NsylCBL5*), and *N. tomentosiformis CBL5* (*NtomCBL5*) (A) and the cloned upstream regulatory region of *NtCBL5A* (B).

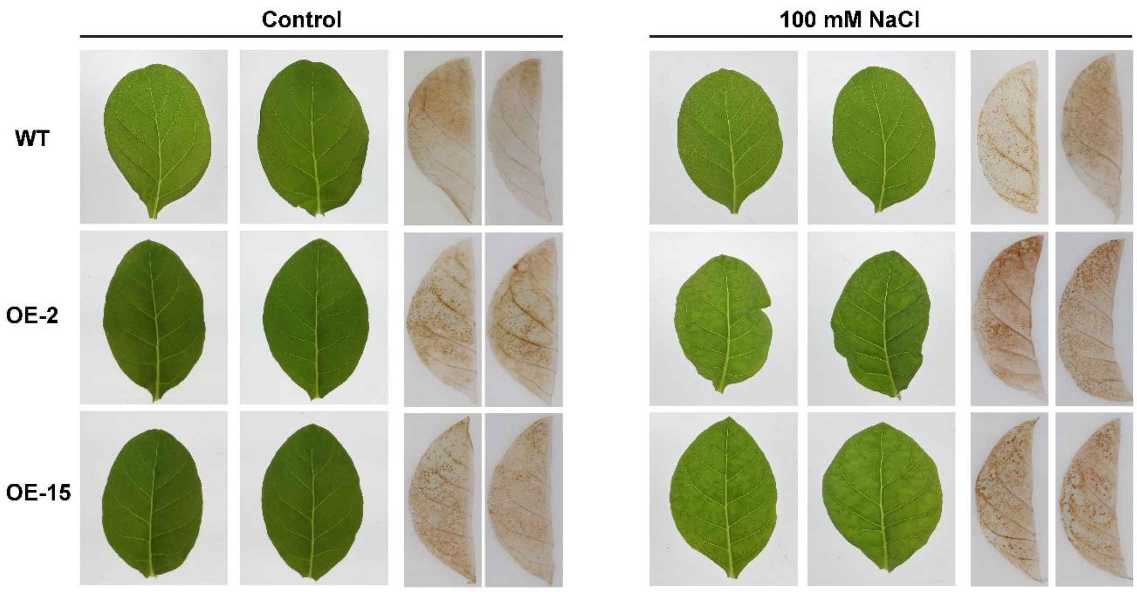

FIGURE S2 | DAB staining of tobacco under control conditions and salt stress (100 mM NaCl) at 2 DAT.

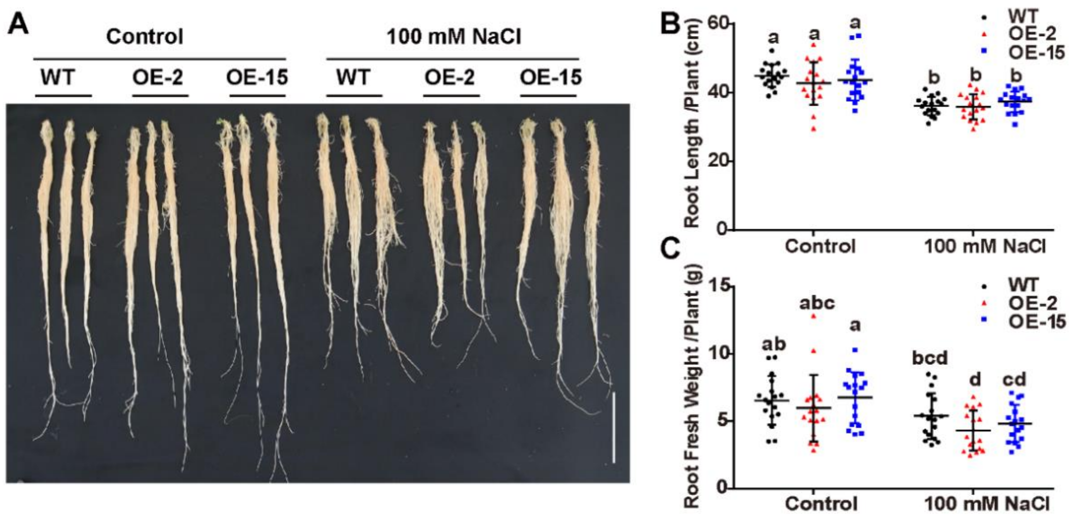

FIGURE S3 | Root phenotype (A), root length (B), and root fresh weight (C) of wild-type (WT) and *NtCBL5A*-overexpressing lines (OE-2 and OE-15) under control conditions and salt stress (100 mM NaCl) at 9 DAT. Scale bar=10 cm. Error bars indicate ±SD (n=17), different letters above bars (a, b, and c) indicate significant statistical difference based on one-way ANOVA with LSD test (P<0.05).

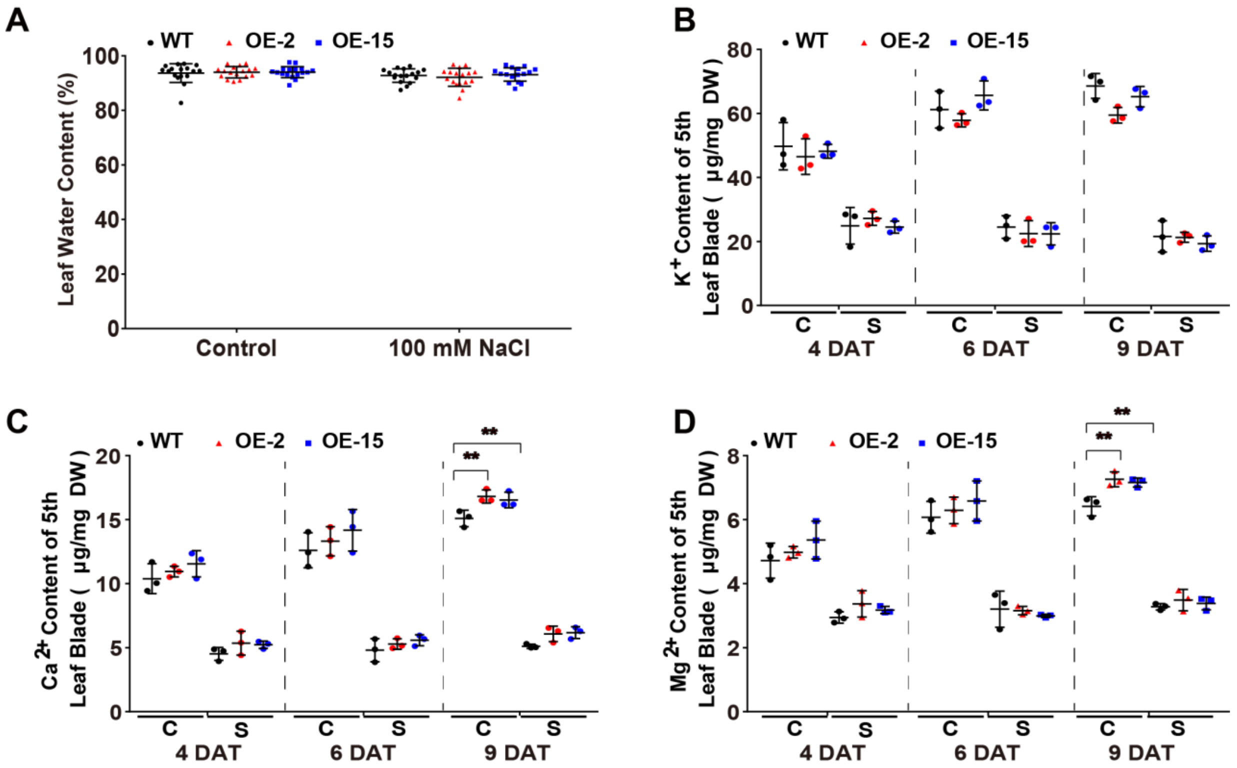

FIGURE S4 | Leaf water content and K^+ , Ca^{2+}, Mg^{2+} determination. (A) Leaf water content of wild-type (WT) and *NtCBL5A*-overexpressing lines (OE-2 and OE-15) under control conditions and salt stress (100 mM NaCl) at 9 DAT. Leaf water content (%) =(W_{fresh}-W_{dry})/W_{fresh}*100% (W_{fresh} is the leaf fresh weight and W_{dry} is the leaf dry weight). Error bars indicate ±SD (n=17). **(C-D)** K^+, Ca^{2+}, and Mg^{2+} contents in the 5th leaf (without main vein) of WT and *NtCBL5A*-OE lines under control conditions and salt stress (100 mM NaCl) at 4 DAT, 6 DAT, and 9 DAT. C means control conditions, while S means salt stress. Error bars indicate ±SD (n=3), every biological replication is a mixed pool of 3 plants. One-way ANOVA with LSD test (*P<0.05 and **P<0.01) was used to analyze statistical significance.

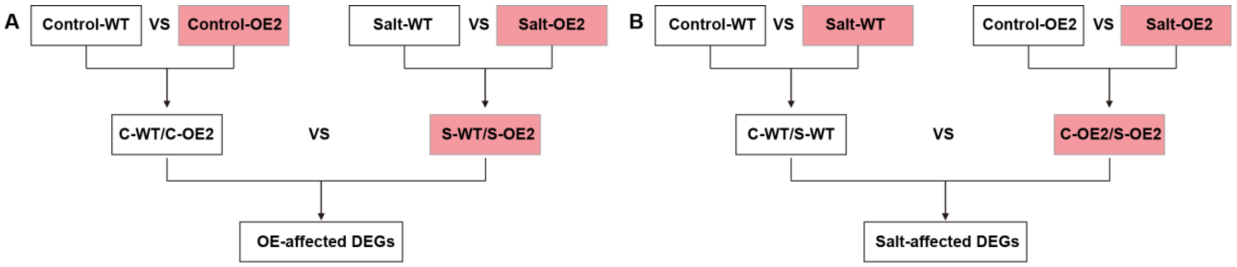

FIGURE S5 | The flow of OE-affected DEGs and Salt-affected DEGs analysis. The read color indicates the “Treatment Group” relative to “Control Group” for transcriptome analysis.

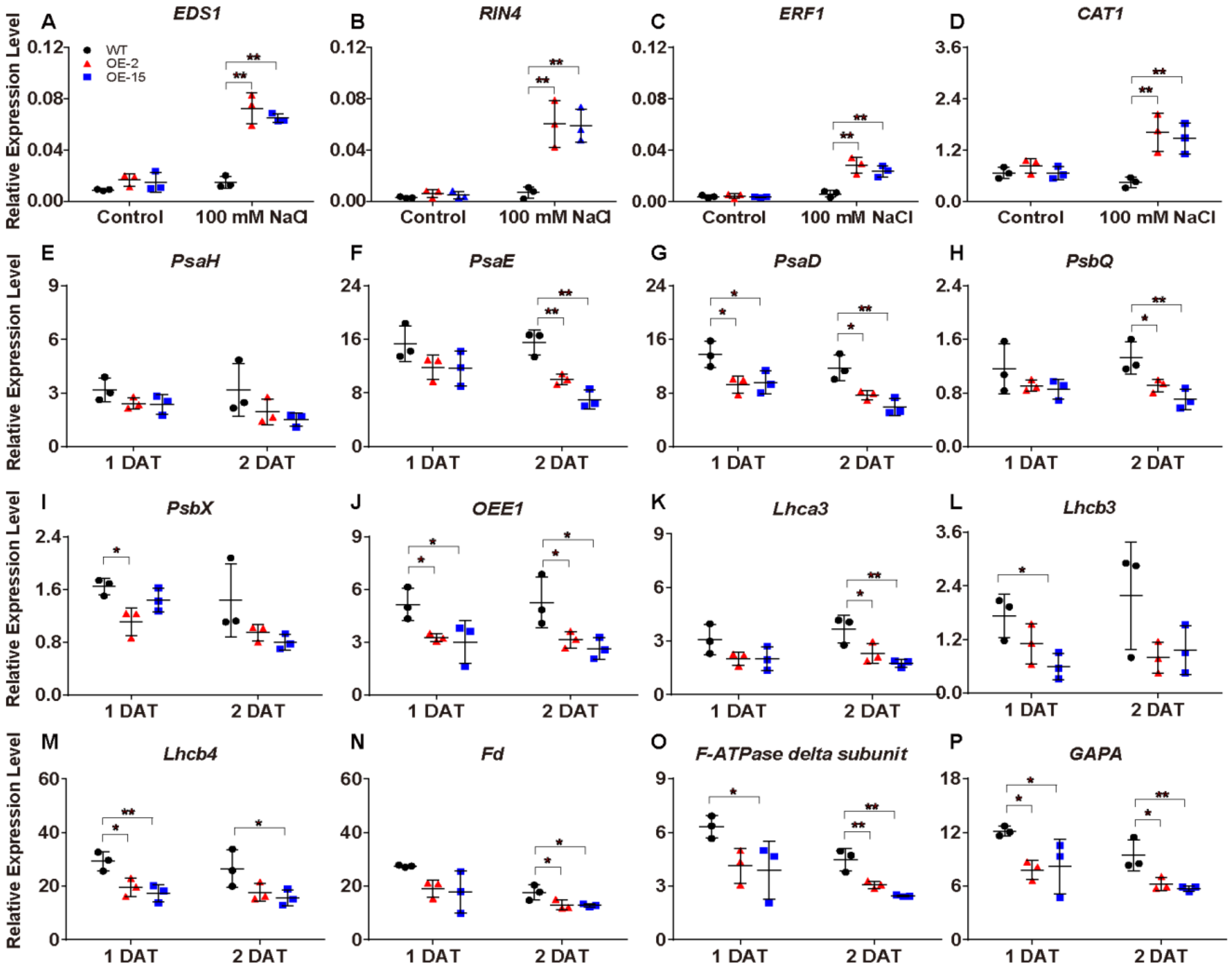

FIGURE S6 | Relative expression analysis of immunity-related genes and photosynthesis-related genes to reference gene *L25* by RT-qPCR. (A-D) Gene expression analysis of immunity-related genes at 4 DAT under control conditions and salt stress (100 mM NaCl). **(E-P)** Gene expression analysis of photosynthesis-related genes at 1 DAT and 2 DAT under salt stress (100 mM NaCl). Their gene IDs in the reference tobacco genome database (ftp://ftp.solgenomics.net/genomes/Nicotiana_tabacum/edwards_et_al_2017/assembly/Nitab-v4.5_genome_Chr_Edwards2017.fasta.gz) are *EDS1* (Nitab4.5_0002101g0050), *RIN4* (Nitab4.5_0003020g0010), *ERF1* (Nitab4.5_0010541g0010), *CAT1* (Nitab4.5_0009821g0010), *PsaH* (Nitab4.5_0000351g0060), *PsaE* (Nitab4.5_0000385g0230), *PsaD* (Nitab4.5_0014875g0010), *PsbQ* (Nitab4.5_0002345g0070), *PsbX* (Nitab4.5_0000073g0060), *OEE1* (Nitab4.5_0000108g0110), *Lhca3* (Nitab4.5_0000923g0200), *Lhcb3* (Nitab4.5_0012832g0010), *Lhcb4* (Nitab4.5_0011597g0020), *Fd* (Nitab4.5_0004129g0010), *F-ATPase delta subunit* (Nitab4.5_0006745g0030), *GAPA* (Nitab4.5_0010299g0040). Error bars indicate ±SD (n=3). One-way ANOVA with LSD test (*P<0.05, **P<0.01) was used to analyze statistical significance.
